# Assessment of Equations to Predict Body Weight and Chemical Composition in Growing/Finishing Cattle and Effects of Publication Year, Sex, and Breed Type on the Deviation from Observed Values

**DOI:** 10.3390/ani12243554

**Published:** 2022-12-15

**Authors:** P. A. Lancaster

**Affiliations:** Beef Cattle Institute, Kansas State University, Manhattan, KS 66506, USA; palancaster@vet.k-state.edu; Tel.: +1-785-532-6323

**Keywords:** beef cattle, carcass, empty body, fat, protein

## Abstract

**Simple Summary:**

Accurate and precise estimation of changes in body weight and chemical composition (i.e., water, fat, protein, and ash) are necessary to determine nutrient requirements of cattle. Many of the methods to measure and equations relating different measures of body weight and different chemical components of the body were established over 40 years ago, but beef cattle genetics and production systems have changed substantially in the last 40 years. Additionally, published equations do not account for sex or breed differences. The current study analyzed published equations using body weight and chemical composition data over the last 40 years from different sexes and breed types. There are differences in the accuracy and precision of the different published equations and no one equation or set of equations was best for all situations (sex, breed type, production system). Additionally, all equations poorly predicted some chemical components (protein and ash) of the body. The results of this analysis indicate that estimation of changes in body weight and chemical composition could be improved by including other factors such as sex and breed type, and that prediction equations may need to be re-evaluated periodically as cattle genetics and production systems change.

**Abstract:**

Body weight and chemical composition are important aspects of beef cattle nutrition and management; however, existing equations estimating relationships among empty body and carcass chemical components were developed over 40 years ago using different cattle genetics and production systems. The objective of this analysis was to evaluate existing equations in predicting empty body and carcass chemical composition and determine the effect of sex, breed type, and publication year. A dataset was developed from published literature that contained 388 treatment means from 46 studies published between 1970 and 2020. Two equations relating shrunk body weight (SBW) to empty body weight (EBW), and 8 equations relating EBW and hot carcass weight (HCW) were found in the literature and evaluated using the developed dataset. Three sets of equations relating empty body chemical components, 4 sets of equations relating carcass chemical components, and 2 sets of equations relating carcass with empty body chemical components were found in the literature and evaluated using the dataset. Precision and accuracy of the equations were evaluated by simple linear regression of observed on predicted values, mean bias (MB), and concordance correlation coefficient (CCC). Additionally, the fixed effects of publication year, sex, and breed type on the deviation from observed values were evaluated using a general linear model. Both equations relating SBW to EBW and all equations relating EBW to HCW had high precision, but accuracy varied from −3.22 to −0.11% and −9.35 to −3.73% MB, respectively, and all the equations were affected by sex and breed type with 8 out of the 10 equations affected by publication year. For prediction of empty body chemical composition assuming empty body water is known, the 3 sets of equations varied in precision for protein (0.18 to 0.46), but not for fat (0.88 to 0.96) or ash (0.06 to 0.13) based on CCC, although the precision of prediction of protein and ash were poor. Accuracy of the 3 sets of equations varied for predicting empty body fat, protein, and ash with MB of −19.73 to −3.81, 1.67 to 15.91, and −0.16 to 15.75%, respectively. All 3 sets of equations were affected by publication year and breed type for predicting empty body fat, protein, and ash, and by sex for ash. For prediction of carcass chemical components assuming carcass water is known, the precision was similar among the 4 sets of equations for predicting fat (0.92 to 0.95), protein (0.34 to 0.40), and ash (−0.02 to −0.01) based on CCC, although precision was poor for protein and ash, but accuracy varied for prediction of carcass fat, protein and ash with MB of −11.20 to −2.52, 2.72 to 8.92, and −4.66 to 20.12%, respectively. Publication year and breed type affected the prediction of carcass fat and protein, and publication year, sex, and breed type affected the prediction of carcass ash for all 4 sets of equations. The precision of predicting empty body chemical components assuming carcass chemical components are known was high for water (0.96 and 0.98), fat (0.97 and 0.98), protein (0.97 and 0.97), and ash (0.98 and 0.96) and similar between the 2 sets of equations based on CCC. The accuracy of predicting empty body water (−1.68 and −0.33%), fat (6.38 and 2.70%), protein (0.85 and −0.54%), and ash (−0.65 and −4.54%) was moderate to high, but differed between sets of equations for fat and ash. Publication year influenced the prediction of empty body water for both sets of equations and ash for one of the equations, whereas, breed type influenced the prediction of water, protein, and ash, but not fat for both equations. Overall, existing equations may have major limitations to predicting empty body protein and ash unless carcass protein and ash are known. Additionally, all the equations were affected by some combination of publication year, sex, and breed type for one or more chemical components. Thus, a more robust set of equations should be developed to account for sex, breed type, and more recent cattle genetics and management systems.

## 1. Introduction

Body weight and chemical composition are instrumental in determining nutrient requirements, estimating rate of gain, and predicting feed intake of growing and finishing cattle [1,2,3]. Body composition can affect energy requirements for maintenance [4], especially visceral organ mass [5,6,7,8]. Additionally, body chemical composition can impact energy requirements for maintenance as the energy required to maintain a gram of chemical fat is from a practical aspect zero (−0.003 kcal/g) but to maintain a gram of chemical protein is on average 0.245 kcal [9,10,11,12,13].

In earlier versions of the Nutrient Requirements of Beef Cattle [14,15] separate equations to predict rate of gain were used for calves vs. yearlings, steers vs. heifers, and medium vs. large frame size due to differences in composition of gain at a given body weight, which impacts the relationship between retained energy and weight gain. In later versions [16,17], the equation predicting rate of gain uses equivalent shrunk body weight, which is body weight adjusted for body composition to a standard reference animal, to achieve a constant relationship between retained energy and weight gain. Of the chemical components of the body, fat is the most critical for estimating retained energy due to the caloric content being almost twice that of protein (9.37 to 9.55 vs. 5.33 to 5.76 kcal/g) [18,19,20,21,22].

The latest version of the Nutrient Requirements of Beef Cattle [16,17] provides adjustments to feed intake prediction equations based on body fat, and although crude adjustments, indicate the influence of body chemical composition on feed intake. At approximately 350 kg equivalent weight, feed intake begins to decline in relation to degree of maturity [23].

As the beef industry moves toward individual cattle management, the relationships among chemical components of the body become of greater importance. Systems to estimate individual animal growth and feed intake rely heavily on estimates of body chemical composition [1,3,24,25,26,27]. Sorting individual cattle by weight, body composition, and/or genetic markers to better achieve optimal carcass endpoints also relies heavily on estimates of body chemical composition [28,29,30].

Therefore, accurate and precise estimation of body weight and chemical composition is critical in beef cattle nutrition and management. However, existing equations estimating relationships among empty body and carcass chemical components were developed over 40 years ago using different cattle genetics and production systems. The objective of this analysis was to evaluate existing equations in predicting empty body and carcass chemical composition and determine the effect of sex, breed type, and publication year.

## 2. Materials and Methods

No animals were used in this analysis and no Institutional Animal Care and Use Committee approval was required.

### 2.1. Database Creation

A literature search was performed to identify experiments measuring shrunk body weight (**SBW**), empty body weight (**EBW**), and carcass weight (**HCW**) of growing/finishing cattle along with empty body, carcass, and offal chemical composition using PubMed and Google Scholar. Multiple searches were used to find studies with body weight measurements, empty body chemical composition measurements, and carcass chemical composition measurements. Search terms cattle, “empty body weight”, and “shrunk body weight” returned 3 and 92 records in PubMed and Google Scholar (excluding terms sheep, goat, lamb, pig), respectively. Search terms cattle, “empty body weight”, and “carcass weight” returned 8 and 477 records in PubMed and Google Scholar (excluding terms sheep, goat, lamb, pig), respectively. Search terms cattle, “empty body”, and “chemical composition” returned 22 and 677 records in PubMed and Google Scholar (excluding terms sheep, goat, lamb, pig), respectively. Search terms cattle, “carcass fat”, and “carcass protein” returned 19 and 419 records in PubMed and Google Scholar (excluding terms sheep, goat, lamb, pig), respectively. Additionally, studies in the gray literature (theses, dissertations, research reports, etc.) known to the authors, but not returned in a database search, were evaluated. Records that were screened first by title, second by species, breed, and sex, and third by methodology. Records using species other than cattle were excluded. Records on various breeds were included and categorized into beef, indicus, and dairy categories; studies and treatments using breed crosses of these categories were excluded. The beef breed type consisted of Angus, Charolais, Belgian Blue, Gelbvieh, Hereford, Limousin, Piedmontese, and Simmental breeds, indicus breed type included Boran, Brahman, Nellore, and Tuli breeds, and dairy breed type included Friesian, Holstein, and Jersey breeds. Records on growing/finishing bulls, steers, and heifers were included in the dataset, but studies or treatments using mature cows or bulls and treatment means based on mixed sex groups were excluded. Only records using direct measurement of all body components to compute empty body weight and proximate chemical analyses to determine empty body, carcass, and offal chemical composition were included; studies using estimating equations to compute empty body weight and body chemical composition were excluded. The final dataset contained 388 treatment means from 46 studies published between 1970 and 2020. Treatment means were further subdivided into a Weight dataset, Empty Body dataset, Carcass dataset, and Empty Body-Carcass dataset based on the type of data available.

### 2.2. Body Weight Relationships

The Weight dataset contained 388 treatment means from 46 studies published between 1970 and 2020, and was used to evaluate published equations to predict EBW from SBW, and HCW from EBW. Shrunk body weight is defined as live body weight following overnight feed withdrawal. Empty body weight is defined as live body weight minus gastrointestinal tract contents. Hot carcass weight is defined as the weight of the carcass after evisceration but prior to cooling. A literature search found 10 published equations relating SBW, EBW, and HCW [17,21,22,31,32,33,34,35]. Two equations were used to compute EBW from SBW: (1) Gil et al. [33]; **Gil1970** and (2) NASEM [17]; **NRC2016**. Eight equations were used to compute HCW from EBW: (1) Lofgreen et al. [31]; **Lofgreen1962**, (2) Garrett and Hinman [21]; **GH1969**, (3) equation 1 of Holzer and Levy [32]; **HL1969_1**, (4) equation 2 of Holzer and Levy [32]; **HL1969_2**, (5) Gil1970, (6) Alhassan et al. [34]; **Alhassan1975**, (7) Ferrell et al. [22]; **Ferrell1976**, and (8) Fox et al. [35]; **Fox1976**. Equations are presented in Table S1.

### 2.3. Empty Body Chemical Composition Relationships

The Empty Body dataset contained 223 treatment means from 30 studies published between 1976 and 2020, and was used to evaluate published equations relating chemical components of the empty body. Observed fat-free dry matter was computed as 100 minus observed empty body water and fat percentage. Observed empty body energy was computed using the reported energy concentrations for fat and protein and the observed empty body fat and protein percentages. A literature search found 3 published sets of equations relating empty body chemical components [21,22,33]. Each set of equations was used to compute empty body chemical composition (fat, fat-free dry matter, protein, ash, and energy): (1) GH1969, (2) Gil1970, and (3) Ferrell1976. Computation of predicted chemical components started assuming empty body water was known to predict empty body fat, then empty body fat-free dry matter, protein and ash were sequentially computed from predicted values. Predicted empty body fat-free dry matter was computed as 100 minus observed empty body water and predicted empty body fat percentages. Predicted empty body protein was computed from observed empty body water or predicted fat-free dry matter using the published equations. Predicted empty body ash was computed as 100 minus observed empty body water and predicted empty body fat and protein percentages. Empty body energy was predicted using two methods: (1) predicted from observed empty body fat percentage using the published equations (**Energy1**), and (2) computed from predicted empty body fat and protein percentages using the reported energy concentrations for fat and protein (**Energy2**). Equations are presented in Table S2.

### 2.4. Carcass Chemical Composition Relationships

The Carcass dataset contained 127 treatment means from 15 studies published between 1976 and 2020, and was used to evaluate published equations relating chemical components of the carcass. Observed fat-free dry matter was computed as 100 minus observed carcass water and fat percentages. Observed carcass energy was computed using the reported energy concentrations for fat and protein and the observed carcass fat and protein percentages. A literature search found 4 published sets of equations relating carcass chemical components [21,22,33,36]. Each set of equations was used to compute carcass chemical composition (fat, fat-free dry matter, protein, ash, and energy): (1) GH1969, (2) Gil1970, (3) Preston et al. [36]; **Preston1974**, and (4) Ferrell1976. Computation of predicted chemical components started assuming carcass water was known to predict carcass fat, then carcass fat-free dry matter, protein and ash were sequentially computed from predicted values. Predicted carcass fat-free dry matter was computed as 100 minus observed carcass water and predicted carcass fat percentages. Predicted carcass protein was computed from observed carcass water or predicted carcass fat-free dry matter using the published equations. Predicted carcass ash was computed as 100 minus observed carcass water and predicted carcass fat and protein percentages. Carcass energy was predicted using two methods: (1) predicted from observed carcass fat percentage using the published equation (**Energy1**), and (2) computed from predicted carcass fat and protein percentages using the reported energy concentrations for fat and protein (**Energy2**). Equations are presented in Table S3. 

### 2.5. Relationships between Carcass and Empty Body Composition

The Empty Body-Carcass dataset contained 101 treatment means from 12 studies published using steers only between 1981 and 2020, and was used to evaluate published equations relating chemical components (water, fat, fat-free dry matter, protein, ash, and energy) of the carcass to the same chemical component of the empty body. Observed empty body, carcass, and offal fat-free dry matter were computed as 100 minus observed water and fat percentages. Observed empty body, carcass, and offal energy were computed using the reported energy concentrations for fat and protein and the observed empty body, carcass, and offal fat and protein percentages, respectively. A literature search found 2 published sets of equations relating carcass and empty body chemical composition [21,22]. Each set of equations was used to compute empty body chemical components assuming all carcass chemical components were known: (1) GH1969 and (2) Ferrell1976. Empty body chemical components were predicted from the corresponding carcass chemical component. Predicted empty body fat-free dry matter was computed as 100 minus predicted empty body water and fat percentages. Equations are presented in Table S4.

### 2.6. Statistical Analyses

Evaluation of published equations to predict body composition was performed using R statistical software (version 4.0.4) with published functions and packages (https://www.rdocumentation.org/ (accessed on 1 December 2022)). Linear regression of observed values on predicted values was performed using the *lm* function of the base statistical package. The model was considered significant at *p* ≤ 0.05. The intercept and slope of the linear regression model were tested equal to zero and one, respectively, using 95% confidence intervals, and the linear hypothesis test simultaneously testing intercept equal to zero and slope equal to one was performed using the *linearHypothesis* function in the *car* package. The mean bias (**MB**), concordance correlation coefficient (**CCC**), and bias correction factor (**Cb**) were computed between observed and predicted values using the *epi.ccc* function in the *epiR* package. The root mean square error of prediction (**RMSEP**) and the coefficient of model determination (**CD**) were computed according to Tedeschi [37].

Factors influencing the deviation between observed and predicted values were identified using the *lm* function in the base statistical package and *Anova* function in the *car* package. The model included fixed effects of publication year, sex, and breed type, where publication year was a continuous variable and breed type and sex were categorical variables. For prediction of empty body and carcass chemical composition independently, observed EBW and HCW, respectively, were used as a covariate to evaluate bias at extreme body weights. For prediction of empty body chemical composition from carcass chemical composition, offal chemical composition was used as a covariate, and the effect of sex was not evaluated because all studies were based on steers. Least square means for categorical variables of sex and breed type were computed using the *emmeans* function in the *emmeans* package. Additionally, least square means were computed at the minimum and maximum values of covariates to visualize differences. Differences among least square means were considered significant at *p* ≤ 0.05 with tendencies at 0.05 < *p* ≤ 0.10.

## 3. Results

### 3.1. Body Weight Relationships

Mean (SD) shrunk, empty, and carcass weight were 373 (145), 338 (135), and 221 (95) kg, respectively, (Table 1) and were strongly correlated (>0.98). For predicting EBW, both equations, Gil1970 and NRC2016, had large coefficients of determination, but the intercept of linear regression was closer to zero for Gil1970 than NRC2016 (Table 2). The slope of linear regression was different from one for NRC2016, but not for Gil1970. Thus, the linear hypothesis test indicated that neither equation met the criteria for unity. However, the MB was greater for Gil1970 than NRC2016 (−3.22 vs. −0.11%, respectively). The RMSEP and Cb, which was very near one, was similar for both equations, but the CD was closer to one for Gil1970 than NRC2016. 

Sex and breed type accounted for significant variation in the deviation between observed and predicted EBW for both Gil1970 and NRC2016, and publication year accounted for significant variation for NRC2016 explaining 27 and 32% of the variation for Gil1970 and NRC2016, respectively. Based on F-values of the ANCOVA, NRC2016 was more affected by sex and breed type than Gil1970. For Gil1970, the equation overpredicted EBW for steers and heifers and underpredicted EBW for bulls with a greater deviation from zero for steers and heifers than bulls. In contrast, NRC2016 underpredicted EBW for bulls to a greater degree than the overprediction for steers and heifers. The Gil1970 equation overpredicted EBW for all breed types, but to a greater degree in dairy type cattle. In contrast, NRC2016 overpredicted EBW in dairy breed types, but underpredicted EBW in indicus and beef breed types.

The prediction of HCW from EBW had a strong coefficient of determination (0.986) for all equations, but the intercept of linear regression was different from zero for all equations except Gil1970 and the slope of linear regression was different from one for all equations except Lofgreen1962 and HL1969_1 (Table 3). Thus, the linear hypothesis test indicated a difference from unity for all equations. The CCC was high (>0.95) for all equations, but Ferrell1976 had the MB closest to zero (−0.84%). The RMSEP was larger for HL1969_2 and Alhassan1975 than the other equations, but the Cb was high for all equations. The CD was 1.00 for Gil1970, whereas the other equations were less than one with Lofgreen1962 being the next closest to one.

Publication year accounted for a significant amount of variation in the deviation between observed and predicted HCW for all equations except Alhassan1975, and sex and breed type accounted for significant variation for all equations explaining 12 (Alhassan1975) to 24% (Gil1970) of the variation. Based on F-values, publication year affected Lofgreen1962, HL1969_1, and Gil1970 more than GH1969 and Alhassan1975 with the other equations in between. For all equations, the deviation shifted toward underprediction with increasing publication year. The HCW of steers had greater underprediction than bulls with heifers being not different from steers or bulls for Lofgreen1962, HL1969_1, and Gil1970. For HL1969_2 and Fox1976, underprediction of HCW was greater for steers and heifers than bulls, and the overprediction of HCW was lesser for steers and heifers than bulls for GH1969, Alhassan1975, and Ferrell1976. Breed type had varying effects on the deviation between observed and predicted HCW among equations. Beef breed types had greater underprediction than dairy which was greater than indicus for Lofgreen1962. For GH1969, overprediction was greater for indicus breed types than dairy breed types with beef breed types being intermediate. Dairy and beef breed types had greater underprediction for HL1969_1, HL1969_2, Gil1970, and Fox1976, and lesser overprediction for Ferrell1976 than indicus breed types. For Alhassan1975, indicus and beef breed types had greater overprediction than dairy breed types. It appears that Gil1970 and Ferrell1976 were the least affected by sex and breed type based on the average absolute deviation.

### 3.2. Empty Body Chemical Composition Relationships

Mean (SD) empty body water, fat, protein, and ash were 59.49 (6.41), 18.03 (7.82), 18.16 (1.95), and 4.33 (1.11) % of EBW, respectively. Mean (SD) empty body protein and ash were 80.83 (4.66) and 19.23 (4.60) % of fat-free dry matter, respectively, ranging from 70.00 to 94.32 % for protein and 6.42 to 30.25 % for ash (Table 4). For prediction of empty body fat from empty body water, the intercept of simple linear regression was different from zero for Gil1970 and Ferrell1976, but not for GH1969; however, the slope of linear regression was different from one for all equations resulting in the linear hypothesis test indicating lack of unity for all equations (Table 5). The CCC was least and MB the greatest for Gil1970 and similar between GH1969 and Ferrell1976. The RMSEP was greater, Cb lesser, and CD further from one for Gil1970 than GH1969 and Ferrell1976.

The deviation between observed and predicted empty body fat was affected by publication year and breed type for all equations, and by EBW for Gil1970 and Ferrell1976 but not GH1969; sex did not influence the deviation for any equation. Publication year, EBW, sex, and breed type explained 27, 28, and 33% of the variation for GH1969, Ferrell1976, and Gil1970, respectively, but all equations were similarly affected by publication year and breed type based on F-values. For both Gil1970 and Ferrell1976, empty body fat was overpredicted to a greater degree at heavier EBW. For all equations, empty body fat was overpredicted to a greater degree in more recent publication years. Dairy and beef breed types were overpredicted by all equations compared to lesser overprediction (Gil1970) or underprediction (GH1969 and Ferrell1976) for indicus breed types.

The prediction of fat-free dry matter was poor for all equations with coefficient of determination of 0.29, CCC ≤ 0.50, and intercept and slope of linear regression different from zero and one, respectively. The CCC was least and MB greatest for Gil1970 and similar between GH1969 and Ferrell1976. The Gil1970 equation also had the intercept furthest from zero and slope furthest from one compared with GH1969 and Ferrell1976. Additionally, Gil1970 had the largest RMSEP, least Cb, and CD furthest from one of all equations.

Similar to empty body fat, the deviation between observed and predicted empty body fat-free dry matter was affected by publication year and breed type for all equations, and by EBW for Gil1970 and Ferrell1976 but not GH1969; sex did not influence the deviation for any equation. These factors explained 27, 29, and 33% of the variation in the deviation for GH1969, Ferrell1976, and Gil1970, respectively. Empty body fat-free dry matter had greater underprediction at heavier EBW for Gil1970 and Ferrell1976, and greater underprediction in more recent publication years for all equations. Dairy and beef breed types had greater underprediction of empty body fat-free dry matter than indicus breed types for all equations.

The prediction of empty body protein was poor for all equations (Table 6). The intercept of linear regression was different from zero and the slope of linear regression was different from one for all equations, but were relatively similar among equations. Thus, the linear hypothesis test indicated a lack of unity for all equations. The CCC and Cb were least and MB greatest for Gil1970 and similar between GH1969 and Ferrell1976. The CD was closest to one for Ferrell1976 and furthest from one for GH1969.

Publication year and breed type affected the deviation between observed and predicted empty body protein for all equations similarly based on F-values, whereas EBW and sex did not affect the deviation for any equation. Publication year and breed type explain approximately 20% of the variation in the deviation for all equations. As publication year increased, the underprediction of the deviation became greater for all equations. For all equations, protein was overpredicted (GH1969 and Ferrell1976) or less underpredicted (Gil1970) in indicus than dairy and beef breed types, and beef breed types had greater overprediction than dairy breed types.

Empty body ash was also poorly predicted by all three equations with coefficient of determination of 0.028 and CCC < 0.150. The intercept of linear regression was different from zero for all equations, the slope was different from one for Gil1970 and Ferrell1976, but not GH1969 which was due to a large standard error, and the linear hypothesis test indicated lack of unity for all equations. Additionally, the MB for GH1969 and Gil1970 was large (>15%), but not for Ferrell1976 (<1%). The RMSEP and Cb were larger, and CD closer to one for Gil1970 than GH1969 and Ferrell1976.

Publication year, sex, and breed type affected the deviation between observed and predicted empty body ash for all three equations, and EBW accounted for significant variation for Gil1970 and tended to for Ferrell1976, which explained 25 (GH1969 and Ferrell1976) to 35% (Gil1970) of the variation. Based on F-values, publication year and breed type affected the deviation similarly for all equations, but Gil1970 was affected by sex more than the other equations. The equations of Gil1970 and Ferrell1976 overpredicted empty body ash at lighter EBW and underpredicted at heavier EBW. For all equations, empty body ash was underpredicted to a greater degree in more recent publication years. Empty body ash was underpredicted for steers more than bulls with heifers being similar to steers (Gil1970 and Ferrell1976) or intermediate (GH1969). For GH1969, empty body ash was overpredicted to a lesser degree in beef compared with dairy and indicus breed types, whereas for Gil1970, beef breed types were overpredicted to a lesser degree than indicus breed types with dairy breed types being intermediate. For Ferrell1976, beef breed types were overpredicted which was different than indicus breed types that were underpredicted, and dairy breed types were intermediate. 

Energy predicted from observed fat percentage (Energy1) had a large CCC (>0.98) with slope of linear regression not different from one for both GH1969 and Ferrell1976, and the intercept of linear regression was not different from zero for GH1969, but the intercept was different from zero for Ferrell1976 (Table 7). However, the linear hypothesis test indicated lack of unity for both equations. The MB and RMSEP were small, Cb was large, and CD close to one for both equations indicating similar predictive ability.

The deviation between observed and predicted energy was influenced by publication year and breed type having similar influence on both equations, but EBW and sex did not influence either equation. Publication year and breed type explained approximately 18% of the variation in the deviation between observed and predicted Energy1 for both equations. Empty body energy was underpredicted to a greater degree in more recent publication years for both equations. Both equations overpredicted Energy1 to a greater degree in indicus than dairy breed types, which were overpredicted to a greater degree than beef breed types.

Energy predicted from predicted fat and protein percentages (Energy2) had a large coefficient of determination (>0.97) and CCC (>0.95), but the slope of linear regression was different than one for GH1969, Gil1970, and Ferrell1976. The intercept of linear regression was not different from zero for GH1969, but was for Gil1970 and Ferrell1976. Thus, the linear hypothesis test indicated lack of unity for all equations. The MB and RMSEP were larger and the Cb and CD further from one for Gil1970 than GH1969 and Ferrell1976. 

Publication year accounted for significant variation in the deviation between observed and predicted Energy2 for all equations, and EBW and sex affected the deviation for Gil1970 and Ferrell1976, but breed type did not influence the deviation for any equation. The amount of variation explained by these variables was 27, 29, and 37% for GH1969, Ferrell1976, and Gil1970, respectively. Based on F-values, EBW had a greater influence on Gil1970 than the other equations, whereas publication year and sex affected all equations similarly except that GH1969 was not affected by sex. As EBW increased Gil1970 and Ferrell1976 had greater overprediction of the deviation in Energy2, and all equations had greater overprediction in more recent publication years. For Gil1970, bulls had lesser overprediction than steers and heifers, which were similar.

### 3.3. Carcass Chemical Composition Relationships

Mean (SD) carcass water, fat and protein were 56.35 (7.67), 21.07 (9.10), and 17.62 (2.82) % of HCW, respectively (Table 8). Protein and ash ranged from 58.46 to 93.75 and 6.25 to 37.25 % of fat-free dry matter, respectively. Prediction of carcass fat from known carcass water was precise with coefficient of determination of 0.905 and CCC > 0.90, but only marginally accurate with MB < −5.00% except for Preston1974 which had a MB of −2.52% (Table 9). The intercept and slope of linear regression were different from zero and one, respectively, for all equations resulting in the linear hypothesis test indicating a lack of unity for all equations. The RMSEP was slightly greater for Gil1970 than the other equations, but the Cb and CD were similar among equations. 

For all equations, HCW, publication year, and breed type, but not sex, were significant factors affecting the deviation between observed and predicted carcass fat. These factors explained approximately 60% of the variation in the deviation between observed and predicted carcass fat. Based on F-values, Preston1974 was least affected by HCW but most affected by publication year among the 4 equations. All equations overpredicted carcass fat to a greater degree as HCW became heavier and in more recent publication years. All equations overpredicted carcass fat to a greater degree in beef breed types than indicus breed types with dairy breed types being intermediate. 

Carcass fat-free dry matter was poorly predicted from known carcass water with coefficient of determination of 0.11 and CCC < 0.40, and MB > 5.00% for all equations except Preston1974. For all equations, the intercept and slope of linear regression were different from zero and one, respectively, and the linear hypothesis test indicated lack of unity. The RMSEP was slightly greater and the Cb lesser for Gil1970 than the other equations. The CD was closer to one for GH1969 and Ferrell1976 than for Gil1970 and Preston1974. 

Similar to carcass fat, the deviation between observed and predicted carcass fat-free dry matter was affected by HCW, publication year, and breed type which explained approximately 60% of the variation for all equations. Publication year affected Preston1974 the most and HCW affected Preston1974 the least based on F-values. In contrast to carcass fat, all equations underpredicted fat-free dry matter to a greater degree with increasing HCW and more recent publication years. Additionally, all equations underpredicted carcass fat-free dry matter to a greater degree in beef breed types than indicus breed types with dairy breed types being intermediate. 

Carcass protein was imprecisely predicted by all equations with coefficient of determination of 0.196 and CCC ≤ 0.40, and inaccurately with MB > 5.00% except for Preston1974 (Table 10). All equations had intercept and slope of linear regression different than zero and one, respectively, and lacked unity according to the linear hypothesis test. The RMSEP was slightly greater, but CD was closer to one for Ferrell1976 than the other equations. The Cb was closer to one for GH1969 and Preston1974 than Gil1970 and Ferrell1976. 

The deviation between observed and predicted carcass protein was affected by publication year and breed type, but not HCW or sex for all equations explaining approximately 35% of the variation. Based on F-values, all equations were similarly affected by publication year and breed type. For all equations, carcass protein was underpredicted to a greater degree in more recent publication years, and overpredicted to a greater degree in indicus breed types than beef and dairy breed types, which were similar.

Predicted carcass ash explained none (r^2^ = 0.00 and CCC < 0.00) of the variation in observed carcass ash for any equation. The GH1969 and Gil1970 equations underpredicted carcass ash on average with MB of 6 and 20%, respectively, whereas Preston1974 and Ferrell1976 overpredicted with MB of −2.1 and −4.6%, respectively. The intercept and slope of linear regression were different than zero and one, respectively, and the linear hypothesis test indicated a lack of unity for all equations. The RMSEP was greater, but the Cb and CD were closer to one for Gil1970 than the other equations.

The deviation between observed and predicted carcass ash was affected by publication year, sex, and breed type for all equations, and by HCW for Gil1970 and Ferrell1976. These factors explained approximately 40 (GH1969, Preston1974, and Ferrell1976) to 48 (Gil1970) % of the variation in the deviation between observed and predicted carcass ash. The equation of Gil1970 was most affected by HCW and least affected by publication year, whereas all equations were affected similarly by sex and breed type based on F-values. Carcass ash was underpredicted to a greater degree with increasing HCW for Gil1970 and Ferrell1976, and in more recent publication years for all equations. For all equations, carcass ash was overpredicted in bulls and underpredicted in steers, and underpredicted in indicus breed types compared with beef and dairy breed types which were similar.

Carcass energy (Energy1 and Energy2) was predicted with high precision (r^2^ > 0.94 and CCC > 0.94) and high accuracy (MB < ±3.0%) except for Gil1970 with a MB of −5.21% for Energy2 (Table 11). The intercept and slope of linear regression were different from zero and one, respectively, for Ferrell1976 for Energy1 and all equations for Energy2, but not for GH1969 for Energy1. However, the linear hypothesis test indicated a lack of unity for all equations for Energy1 and Energy2. The RMSEP, Cb, and CD were similar among equations for Energy1, but for Energy2, Gil1970 had Cb and CD further from one, and RMSEP slightly greater than the other equations.

For Energy1, the deviation between observed and predicted carcass energy was affected by publication year, sex, and breed type, but not HCW for GH1969 and Ferrell1976 which explained approximately 28% of the variation. Both equations were similarly affected by sex and breed type, but Ferrell1976 was more affected by publication year than GH1969 based on F-values. Carcass Energy1 was underpredicted to a greater degree in more recent publication years, underpredicted to a greater degree in bulls than steers, and overpredicted to a greater degree in indicus breed types than beef and dairy breed types, which were similar, for both equations.

For Energy2, HCW, publication year, and sex, but not breed type, affected the deviation between observed and predicted carcass energy for all equations explaining approximately 60% of the variation. Based on F-values, Gil1970 was most affected by HCW and least affected by publication year, and Preston1974 was least affected by HCW and most affected by publication year, whereas all equations were similarly affected by sex. All equations overpredicted carcass Energy2 to a greater degree at heavier HCW, and in more recent publication years. Again, all equations overpredicted carcass energy in steers and underpredicted carcass energy in bulls.

### 3.4. Relationships between Carcass and Empty Body Composition

Mean (SD) carcass water, fat, and protein were 56.81 (8.12), 20.14 (9.15), and 17.87 (2.97) % of HCW, respectively, and empty body water, fat, and protein were 58.05 (6.97), 19.20 (8.01), and 18.51 (2.25) % of EBW, respectively (Table 12). As a percentage of fat-free dry matter, carcass protein ranged from 58.46 to 93.75%, and empty body protein ranged from 70.63 to 93.58%.

Prediction of empty body water from carcass water was precise with coefficient of determination of 0.956 and CCC > 0.96, and accurate with MB < ±2.0% for both equations (Table 13). The intercept and slope of the simple linear regression was different from zero and one, respectively, for GH1969, but not for Ferrell1976. The linear hypothesis test indicated a lack of unity for GH1969, but not for Ferrell1976. The RMSEP was less and CD closer to one for Ferrell1976 than GH1969, and the Cb was nearly perfect (1.00) for Ferrell1976 at 0.999.

The effect of sex was not evaluated in the Empty Body-Carcass dataset because all observations were using steers. The water concentration of offal, EBW, publication year, and breed type explained approximately 30 to 40% of the variation in the deviation between observed and predicted empty body water with these variables explaining more variation for GH1969 than Ferrell1976. Based on F-values, Ferrell1976 was more affected by offal water percentage than GH1969, but both equations were similarly affected by EBW and publication year. Empty body water was underpredicted to a greater degree as offal water increased for Ferrell1976. For both GH1969 and Ferrell1976, empty body water was underpredicted to a greater degree as EBW increased. For both equations, empty body water was underpredicted to a greater degree in more recent publication years. Breed type did not influence prediction of empty body water.

Empty body fat was predicted with high precision having coefficient of determination of 0.961 and CCC > 0.96 for both equations, but Ferrell1976 had better accuracy than GH1969 (MB = 2.70 vs. 6.38%, respectively). Additionally, the intercept and slope of linear regression was not different from zero and one, respectively, for Ferrell1976, but were different for GH1969. However, the linear hypothesis test indicated lack of unity for both equations. The RMSEP was less, and the Cb and CD were closer to one for Ferrell1976 than GH1969. 

The deviation between observed and predicted empty body fat was affected by offal fat percentage for Ferrell1976, and EBW for GH1969, but was not affected by publication year or breed type for GH1969 and Ferrell1976, which explained 12 and 15% of the variation for GH1969 and Ferrell1976, respectively. Empty body fat was underpredicted to a greater degree with increasing offal fat percentage for Ferrell1976, and was overpredicted to a greater degree with increasing EBW for GH1969.

Empty body fat-free dry matter was predicted with high precision with coefficient of determination of 0.837 and 0.886, and CCC of 0.906 and 0.931, and with high accuracy having MB −1.1 and −1.4% for GH1969 and Ferrell1976, respectively. The intercept of linear regression was different from zero for GH1969, and slope was different from one for GH1969 and Ferrell1976. The linear hypothesis test indicated a lack of unity for both equations. The RMSEP and Cb were lesser, but CD greater for Ferrell1976 than GH1969. 

Offal fat-free dry matter percentage and publication year, but not EBW or breed type, significantly influenced the deviation between observed and predicted empty body fat-free dry matter for both equations explaining 60 and 54% of the variation for GH1969 and Ferrell1976, respectively. The F-value for offal fat-free dry matter was larger for Ferrell1976 than GH1969, but for publication year the F-value was larger for GH1969 than Ferrell1976 indicating greater influence on the deviation between observed and predicted values. For both equations, empty body fat-free dry matter was underpredicted to a greater extent with increasing offal fat-free dry matter percentage, and was overpredicted to a greater extent in more recent publication years. 

Empty body protein was predicted with high precision having coefficient of determination of 0.944 and CCC of 0.96, and high accuracy with MB of 0.85 and −0.54% for GH1969 and Ferrell1976, respectively (Table 14). The intercept and slope of linear regression were different from zero and one, respectively, for both equations, and the linear hypothesis test indicated lack of unity for both equations. The RMSEP was low and Cb was high and both were similar between equations, but the CD was closer to one for GH1969 than Ferrell1976. 

Percentage of protein in offal, but not EBW, publication year, or breed type, accounted for significant variation in the deviation between observed and predicted empty body protein for both equations explaining 23 and 14% of the variation for GH1969 and Ferrell1976, respectively. Based on F-values, offal protein percentage had more influence on the deviation for GH1969 than Ferrell1976. For both equations, the deviation between observed and predicted protein was underpredicted to a greater degree as offal protein percentage increased. 

Empty body ash was predicted with high precision with coefficient of determination of 0.958 and CCC > 0.95 for both equations, and with high accuracy for GH1969 with MB of −0.65%. The intercept and slope of linear regression was not different from zero and one, respectively, and the linear hypothesis test indicated unity for GH1969, but the intercept and slope were different from zero and one, respectively, for Ferrell1976. The RMSEP was less and Cb was greater for GH1969 than Ferrell1976, but the CD was similar.

The deviation between observed and predicted empty body ash was influenced by offal ash percentage and breed type for both equations, and by publication year for GH1969 explaining 87 and 53% of the variation for GH1969 and Ferrell1976, respectively. The F-values for offal ash percentage and publication year were larger for GH1969 than Ferrell1976, but the F-value for breed type was larger for Ferrell1976 than GH1969. Empty body ash was underpredicted to a greater degree as offal ash percentage increased for both equations and underpredicted to a greater degree in more recent publication years for GH1969. For GH1969, ash was overpredicted more in indicus than beef breed types with dairy breed types being intermediate, whereas, for Ferrell1976, indicus breed types were more overpredicted than beef and dairy breed types which were similar.

Both equations predicted empty body energy with high precision having coefficient of determination of 0.963 and CCC > 0.96, but Ferrell1976 predicted energy with more accuracy (MB = 2.17 vs. 4.33%) than GH1969. The intercept and slope of linear regression were not different from zero and one, respectively, for Ferrell1976, but were for GH1969. However, the linear hypothesis test indicated lack of unity for both equations. The RMSEP and Cb were similar between equations, but Ferrell1976 had CD closer to one than GH1969.

Offal energy concentration and EBW affected the deviation between observed and predicted empty body energy for both equations explaining 22 and 25% of the variation for GH1969 and Ferrell1976, respectively. Based on F-values, Ferrell1976 was more influenced by offal energy concentration than GH1969, whereas GH1969 was more influenced by EBW than Ferrell1976. Energy was underpredicted to a greater degree as offal energy concentration increased, and overpredicted to a greater degree as EBW increased for both equations. Publication year and breed type did not influence the prediction of energy. 

## 4. Discussion

### 4.1. Relationship among Body Weights

The precision among SBW, EBW, HCW is high, and the relationship is near 1, but the accuracy is variable with large intercepts and MB. The NRC2016 uses a constant proportion (0.891) of SBW to estimate EBW, whereas Gil1970 uses an equation with slope less than one and negative intercept suggesting that EBW is not a constant proportion of SBW, which is supported by the simple linear regression slope of one for Gil1970 versus a slope greater than one for NRC2016. Additionally, Williams et al. [38], Owens et al. [39], and Owens and Hicks [40] demonstrate that EBW is not a constant proportion of SBW, and that the difference (i.e., gut fill) depends upon several dietary factors and age and weight of the cattle where lighter calves consume more feed as a percentage of SBW than heavier yearlings [17,41].

Similarly, many of the equations to predict HCW from EBW had simple linear regression slopes less than one and positive intercepts indicating that carcass is a lesser proportion of EBW at lighter body weights. This is supported by the slope coefficients of published equations being greater than one [39]. Additionally, previous research indicates that carcass weight is a greater proportion of live weight with increasing live weight and days on feed [42,43,44,45,46], which is supported in our data where dressing percentage increased 2 percentage units for each 100 kg increase in SBW.

Publication year affected many of the equations to predict EBW from SBW and HCW from EBW where EBW and HCW were underpredicted to a greater degree in more recent publication years which indicates that EBW is a greater proportion of SBW and HCW is a greater proportion of EBW in more recent publication years. In our data, the heaviest SBW tended to be in more recent publication years, and EBW and HCW as a proportion of SBW increased as SBW increased. Thus, the effect of publication year may be an indirect effect of SBW.

Empty body weight of bulls was underpredicted compared with steers and heifers suggesting that bulls have greater EBW as proportion of SBW as the published equations were developed using steers and heifers. In our data, EBW (0.93 vs. 0.87 and 0.86) was a greater proportion of SBW in bulls than steers and heifers, respectively, after adjustment for publication year and breed type. However, HCW was overpredicted in bulls compared with steers and heifers suggesting that bulls have lesser HCW as a proportion of EBW, which was found in our data (0.63 vs. 0.65 and 0.64, respectively). Few studies have evaluated the effect of sex on EBW as proportion of SBW or HCW as proportion of EBW, but several have evaluated dressing percentage with mixed results. Hedrick et al. [47] and Nichols et al. [48] reported lesser dressing percentage, Vanderwert et al. [49] reported no difference in dressing percentage, and others [50,51,52,53,54,55,56] have reported greater dressing percentage in bulls than steers and/or heifers particularly at heavier live weight. In our data, dressing percentage (0.58 vs. 0.57 and 0.56) was slightly greater in bulls than steers and heifers, respectively.

The overprediction of EBW in dairy compared with indicus and beef breed types may be due to differences in feed intake and gastrointestinal fill. However, data are conflicting whether Holstein steers eat more [57,58] or the same [59] as beef steers. In a summary of published studies, Rust and Abney [60] reported greater feed intake as a percentage of body weight in Holstein steers compared with Hereford steers, but not Angus steers. Additionally, Holstein steers had greater feed intake compared with beef steers in the VetLife Benchmark data [60].

In contrast to EBW, the MB for dairy breed types was similar to beef breed types for most equations to predict HCW, and the indicus breed types were overpredicted compared with dairy and beef breed types. The difference in breed type rankings between prediction of EBW and HCW is interesting and suggests differences in relationships between gastrointestinal fill and visceral organ mass/offal mass. In our data, dairy had lesser EBW as a proportion of SBW (0.86 vs. 0.91 and 0.91) than indicus and beef breed types, respectively, and similar HCW as a proportion of EBW (0.63 and 0.64 vs. 0.66) to indicus, but less than beef breed types, respectively. However, the magnitude of difference was less for HCW than EBW proportions in dairy compared with other breed types.

Overall mean deviations for sex and breed type are within reasonable limits ranging from −4.6 to 5.2% of EBW with similar magnitude of mean bias between sexes and breed types for both equations. However, the impact on accuracy of predicting HCW could be quite large in some situations (e.g., dairy heifers in recent years using HL1969_2), but minor in other situations (e.g., beef steers in recent years using Ferrell1976). Holzer and Levy [32] did not include kidney, pelvic and trim fat in carcass weights and these equations had the greatest MB as well as the largest effects of publication year, sex, and breed type. Excluding HL1969_1 and HL1969_2, prediction of HCW was within reasonable limits among sexes and breed types having mean deviations ranging from −8.17 to 5.01%. However, none of the equations evaluated would be the single best choice to predict EBW or HCW in all situations.

### 4.2. Relationship among Components of Empty Body and Carcass

For both empty body and carcass composition, the prediction of fat from water percentage was highly precise, although not always highly accurate, for all equations. The coefficients of the published equations are very similar suggesting a tight relationship between body water and fat percentage. Reid et al. [61] reported a strong inverse relationship between empty body water and fat percentage in male and female dairy and beef cattle, which they demonstrated to be curvilinear with extreme empty body fat percentages ranging from 1.8 to 44.6%—much fatter than typical endpoints of cattle fed today. Overall, GH1969 and Ferrell1976 similarly predict empty body fat and appear to better predict empty body fat than Gil1970. There appears to be little difference among equations to predict carcass fat, but Preston1974 is slightly more accurate.

Empty body and carcass fat-free dry matter were predicted with very poor precision, and poor to high accuracy. Predicted fat-free dry matter was computed as 100 minus observed water and predicted fat and such the only difference between observed and predicted fat-free dry matter was predicted fat, which is reflected in the exact same values for RMSE and RMSEP for predicted fat and predicted fat-free dry matter as well as the MB for sex and breed types being the negatives of each other. Thus, all of the error in predicted fat was captured in predicted fat-free dry matter. The correlation of empty body water and fat-free dry matter was 0.54 and between empty body fat and fat-free dry matter was −0.72 indicating a considerable degree of variation in the relationships. Additionally, the correlation of carcass fat-free dry matter with water was 0.33 and with fat was −0.61. Because predicted fat is predicted from observed water, predicted fat accounts for no additional variation in fat-free dry matter only adjusting the mean fat-free dry matter, which was reflected in the high accuracies of some equations, thus fat-free dry matter is essentially being predicted from water which has poor correlation with fat-free dry matter in our dataset. However, Gil et al. [33], Ferrell et al. [22], and Preston et al. [36] reported strong correlations between water and protein in the empty body (0.85 to 0.95) and carcass (0.92 to 0.95). Preston et al. [36] reported a correlation of 0.79 between carcass water and ash. Additionally, the imprecision of predicting fat-free dry matter could be due to differences in the cattle used to develop the equations compared with the current dataset. Our dataset contained data from bulls, steers, and heifers of diverse breed types, whereas the cattle used to develop the equations were steers (GH1969 and Preston1974), heifers (Ferrell1976), or bulls, steers, and heifers (Gil1970) of Hereford (Gil1970, Ferrell1976, and GH1969) or Hereford and Angus breeding (Preston1974). The range of values for empty body water, fat, and protein in our dataset were greater than for GH1969 (water ~ 45 to 64% EBW; fat ~ 13 to 36% EBW; protein ~ 14 to 19% EBW, based on ±2 SD from the mean), Gil1970 (water ~ 49 to 69% EBW; fat ~ 8 to 34% EBW; protein ~ 13 to 18% EBW), and Ferrell1976 (water ~ 49 to 61% EBW; fat ~ 16 to 33% EBW; protein ~ 15 to 18% EBW), and the range of values for carcass water, fat, and protein in our dataset were greater than Preston1974 (water ~ 40 to 61% EBW; fat ~ 15 to 43% EBW; protein ~ 12 to 18% EBW, based on ±2 SD from the mean).

Empty body and carcass protein were predicted with poor precision in all equations, and poor to high accuracy among equations. For Gil1970, Preston1974, and Ferrell1976, protein is predicted from water, which only had a moderate correlation (0.50 and 0.44) with protein in the empty body and carcass, respectively, in our dataset. For GH1969, protein is predicted from fat-free dry matter and thus, some of the poor prediction of protein was due to the poor prediction of fat-free dry matter, which was essentially predicted from water as discussed above. Using observed fat-free dry matter in GH1969 substantially improved the prediction of empty body and carcass protein with CCC of 0.826 and 0.753 and MB of −2.75 and −0.96%, respectively, although not to the level of precision of the relationship between water and fat. Garrett and Hinman [21] reported correlations of 0.99 and 0.98 between fat-free dry matter and nitrogen in the empty body and carcass, respectively, compared to 0.85 and 0.77 in our dataset. In most situations observed fat-free dry matter is not known such as when estimating carcass fat from specific gravity and empty body water from urea space of the body resulting in essentially predicting protein from water as the equations of Gil1970, Preston1974, and Ferrell1976. The principle that protein can be predicted from water or fat-free dry matter is reliant on the assumption that protein is a consistent proportion of the fat-free matter or fat-free dry matter, respectively. Murray [62] and Garrett and Hinman [21] reported protein was approximately 21% of the fat-free matter of the empty body and carcass. In our dataset, mean (SD) protein was 22.30 (1.93) and 22.35 (2.82)% of fat-free matter in the empty body and carcass, respectively, which agrees well with Murray [62] and Garrett and Hinman [21] but the standard deviations are 3 times the standard deviation reported by Garrett and Hinman [21]. Additionally, mean empty body and carcass protein as a percentage of fat-free dry matter in our dataset was similar to Reid et al. [61] and Garrett and Hinman [21] but the standard deviations are 3 times larger. Our dataset has more diversity of breed types and greater ranges in body composition than datasets used to derive equations. Thus, predicting empty body and carcass protein with high precision may be difficult, and requires further study.

Similar to protein, prediction of empty body and carcass ash had very poor precision and in several cases poor accuracy. In fact, the slope for simple linear regression of observed on predicted carcass ash values was not different from zero for all equations. Predicted ash was computed as 100 minus observed water and predicted fat and protein resulting in all the error in predicting fat and protein accumulating in predicted ash. Preston et al. [36] reported a correlation of 0.79 between carcass water and ash, but in our dataset the correlation between carcass water and ash was −0.02 and between empty body water and ash was 0.16. Similar to protein, mean ash as a percentage of fat-free matter in the empty body and carcass in our dataset were similar to Reid et al. [61] and Garrett and Hinman [21] but the standard deviations were 6 times greater. Although ash is probably the least important chemical component in nutritional studies, better knowledge of the relationship of ash with other chemical components is warranted.

Both methods of predicting empty body and carcass energy were highly precise and accurate with little difference between methods except the RMSEP was slightly greater for Energy2. The likely reason for this is due to fat explaining > 95% of the variation in energy concentration, thus, even though protein concentration explains 58% of the residual energy concentration, protein only explains an additional 3% of the total variation in energy. Additionally, empty body and carcass fat were predicted with high precision. With the poor prediction of empty body and carcass protein, predicted protein negatively impacted the precision of predicting energy concentration as r^2^ and CCC were lesser for Energy2 than Energy1. Both Garrett and Hinman [21] and Ferrell et al. [22] reported very strong correlations (0.99) of empty body and carcass energy with water and fat. Given that empty body and carcass fat are predicted with high precision and accuracy, estimating energy retention in nutrition studies from known empty body water or carcass specific gravity is likely to be highly precise and accurate.

#### 4.2.1. Effect of Weight

The overprediction of empty body and carcass fat composition at heavier weights is possibly due to a greater range in body weights and fat percentage in the current dataset than those used to develop the equations, but may also be due to selection for increased muscling of cattle [63]. The effect of increased muscling can be seen in the underprediction of empty body and even more so carcass fat-free dry matter at heavier weights. Interestingly, protein was not affected by empty body or carcass weight, but empty body and carcass ash were underpredicted at heavier weights indicating that ash may be a lesser percentage of body weight at the heavier finishing weights in more recent publication years.

The overprediction of empty body and carcass Energy2 at heavier weights is likely a reflection of the overprediction of fat at heavier weights. The lack of influence of weight on Energy1 is likely due to predicting energy from observed rather than predicted fat percentage. Energy was not predicted from predicted fat percentage alone as this would provide similar results as predicting fat percentage from water as energy is observed fat multiplied by a constant value.

The effect of weight on predicted fat in the carcass (−18.5 to 7.97%) and empty body (−25.5 to 2.4%) was considerable. The magnitude of the deviations could significantly impact estimates of retained energy where initial harvest cattle are considerably lighter than final harvest cattle.

#### 4.2.2. Effect of Publication Year

The use of the equations evaluated in current beef cattle production systems could result in significant errors overpredicting empty body and carcass fat from 2 to 6% units. As discussed above, publication year and body weight are somewhat linked in this dataset, but the effect of publication year is also likely a reflection of changes in cattle genetics and management systems. This suggests that the relationship between water and fat percentage has changed over time, which we expect is a change in the intercept rather than the slope as the displacement of water with fat is consistent among published equations, although we do not have enough data to test this hypothesis. In our dataset, empty body fat decreased (*p* < 0.01) −0.125 ± 0.023% units and carcass fat decreased (*p* < 0.01) −0.260 ± 0.029% units with each publication year after adjusting for weight, sex, and breed type. Additionally, the ratio of empty body fat to empty body water (−0.0023 ± 0.0005 for each year; *p* < 0.01) and carcass fat to carcass water (−0.0046 ± −0.0007 each year; *p* < 0.01) decreased with increasing publication year even after adjusting for fat, weight, sex, and breed type. Selection for increased muscling and finishing cattle at heavier weights while maintaining similar fat composition at finish has likely altered the relationship of water with fat assuming fat-free matter is a relatively constant proportion of water, protein, and ash. Calculated from the data of Ferrell and Jenkins [64], more muscled Belgian Blue and Peidmontese-sired steers (0.50) had lesser ratios of empty body fat to water then less muscled Angus/Hereford-sired steers (0.61). To this point, empty body and even more so carcass fat-free dry matter were underpredicted in more recent publication years indicating a difference in the relationship of water with fat. Additionally, growth promoting implants alter the relationship between fat and water [65,66,67,68] with the more aggressive implants used in more recent publication years having a greater impact [69] which is likely part of the influence of publication year. Empty body and carcass protein and ash follows the same trend as fat-free dry matter likely for the same reasons.

Given that observed empty body and carcass energy is computed from energetic values of fat and protein, the underprediction of Energy1 in more recent publication years indicates that protein is having a greater impact on energy concentration. In contrast to fat, empty body protein increased (*p* < 0.01) 0.054 ± 0.011% units and carcass protein increased (*p* < 0.01) 0.105 ± 0.018% units with each publication year after adjusting for weight, sex and breed type indicating a changing ratio of fat to protein that likely affected the prediction of energy. The influence of publication year on Energy2 is opposite that of Energy1 indicating that the overprediction of fat in more recent publication years is affecting prediction of energy.

The magnitude of the deviations for empty body fat (−29.1 to −13.5%) and protein (5.2 to 19.6%) in more recent publication years could significantly affect estimates of retained energy. Fat was overpredicted indicating that energy estimated from fat alone would be overestimated; however, protein was underpredicted compensating for the overestimation of energy although protein, having 60% the energy value of fat, does not fully compensate.

#### 4.2.3. Effect of Sex

Sex only affected the prediction of empty body ash and carcass ash and energy. The overprediction of ash in bulls compared with steers and heifers suggests that ash is a lesser percentage of weight than in steers and heifers as equations were developed using data from steers or heifers with the exception of Gil1970 which used bulls, steers, and heifers. Interestingly, Gil1970 did not predict ash of bulls, steers, and heifers any more accurately than the other equations. Previous studies indicate that bulls do not have lesser ash [70] or bone [52,71,72] percentage of the empty body or carcass, but in our dataset, bulls had lesser (*p* < 0.05) carcass ash than steers (3.03 vs. 5.49%, respectively), but not empty body ash than steers or heifers (4.02, 4.66, and 4.57%, respectively) after adjustment for weight, publication year, and breed type.

Carcass energy of bulls was underpredicted by observed carcass fat percentage alone most likely due to greater proportion of protein than steers [52,70,71], but prediction of Energy2, which considers predicted fat and protein, was not improved compared with Energy1. The lack of improvement in prediction of energy with inclusion of protein could be due to the poor precision of predicting protein, but sex did not influence prediction of carcass protein.

The magnitude of the effect of sex on empty body and carcass ash was considerable ranging from −2 to +2% units corresponding to approximately a ±50% error. The magnitude of the effect of sex on carcass energy is relatively small with the largest deviation (−0.18) corresponding to a 6.6% error. Thus, sex may not be a major factor in predicting empty body or carcass fat, protein, and energy.

#### 4.2.4. Effect of Breed Type

The underprediction of empty body and carcass fat in indicus breed types compared with beef and dairy breed types is opposite of what might be expected. Indicus breed types have less body fat than British breed types [73,74,75,76,77], but more than Continental breed types [78]. Predictive equations were developed from beef breed types, primarily Hereford, and thus would be expected to overpredict fat percentage in leaner indicus breed types. To this point, fat-free dry matter and protein were overpredicted in indicus compared with dairy and beef breed types. The relationship between water and fat may be different in indicus breed types, and as mentioned above is likely a difference in the intercept rather than the slope.

Differences in mean bias of ash among breed types did not follow a similar pattern in the empty body and carcass. In the carcass, ash percentage of indicus breed types was underpredicted compared with dairy and beef breed types, which is the opposite trend observed with carcass protein suggesting that indicus breed types may have a greater percentage of bone or lesser muscle-to-bone ratio in the carcass. Huffman et al. [79] and Bidner et al. [77] reported greater longissimus muscle area per kg of HCW in Angus than indicus breed types, and Cole et al. [80] reported greater separable bone percentage of the carcass in indicus than beef breed types. However, Lunt et al. [81] reported no difference in separable muscle or bone percentage of the carcass and no difference in the muscle to bone ratio of Angus and Brahman steers. Interestingly, the mean bias of dairy breed types was similar to beef breed types, which is somewhat unexpected as dairy breed types typically have greater percent bone in the carcass [80,82,83,84].

In contrast to the carcass, ash was underpredicted in the empty body of indicus and dairy breed types compared with beef breed types. Ferrell and Jenkins [73] reported similar amounts (kg) of ash that correspond to similar percentages in the carcass (6.2 and 6.2%), offal (2.5 and 2.4%), and empty body (4.9 and 4.9%) of Angus and Brahman steers, respectively. In our dataset, indicus breed types had greater (*p* < 0.05) empty body (5.31 vs. 4.12 and 3.83%) and carcass (5.82 vs. 3.57 and 3.38%) ash than dairy and beef breed types, but offal ash was similar. These results explain similarity in mean bias between dairy and beef breed types for carcass ash, but not the similarity between dairy and indicus in empty body ash. In the empty body, fat was underpredicted and protein overpredicted in indicus compared with dairy breed types likely resulting in similar mean bias as ash was computed as 100 minus observed water and predicted fat and protein, whereas in the carcass the mean bias of protein, but not fat, was different between indicus and dairy breed types.

The overprediction of empty body and carcass energy in indicus compared with dairy and beef breed types when based on observed fat percentage may reflect differences in proportion of fat and protein in the carcass and offal. Several studies have indicated that indicus breed types have less fat and more or similar muscle/protein in the carcass compared with beef breed types [73,77,80,81]. Ferrell and Jenkins [73] reported no difference in fat and protein composition of the offal in indicus and beef breed types suggesting that differences in offal composition is not impacting prediction of empty body energy. The lack of breed type effect when energy is predicted from fat and protein supports the idea that indicus cattle have a difference in fat to protein ratio compared with beef and dairy breed types.

### 4.3. Relationship of Empty Body with Carcass Composition

Overall, the prediction of empty body chemical components from known carcass chemical components was highly precise and accurate indicating that if carcass composition is measured via proximate analysis, empty body composition can be predicted. In general, the mean bias of empty body chemical components was less than when predicting empty body composition from known empty body water. Only empty body fat using GH1969 had what might be consider unacceptable mean bias. Additionally, the amount of variation in the deviation explained by offal chemical composition, publication year, sex and breed type was low for fat, protein, and energy, high for fat-free dry matter and ash, and intermediate for water. The most likely reason for deviation between the carcass and empty body is chemical composition of the offal, and offal chemical components generally had the largest F-values in models to explain the deviation. Offal water, fat, protein, and ash in our dataset was highly variable (coefficient of variation = 11.22, 43.03, 9.02, and 37.45%, respectively).

Offal protein was the only significant factor explaining variation in the deviation between observed and predicted empty body protein. Based on data from Coleman et al. [85], Hersom et al. [86], and McCurdy et al. [87] offal protein as a percentage of total empty body protein decreases with increasing weight, whereas, carcass protein increases, which explains the over and underprediction at low and high percentages of offal protein, respectively. Thus, more precise prediction of empty body protein will require development of equations to predict variation in offal protein. Mass of visceral and splanchnic tissues is likely the most variable component of offal protein [8,88], and is the most important from an energy expenditure standpoint [4]. Visceral and splanchnic tissues vary with nutrition [5,8,89,90,91,92,93] and breed [64,73,90,94]. Developing equations to predict mass of visceral and splanchnic tissues would improve predictions of empty body protein and estimates of maintenance energy requirements.

Generally, empty body composition was overpredicted at lower offal chemical concentration and underpredicted at greater offal chemical concentration, which is logical if accurately predicting the mean; however, it indicates a lack of adjusting for the changing chemical composition and relative proportion of offal chemical components in the empty body. Growth coefficients of different fat depots suggest that carcass fat becomes a greater percentage of total body fat as weight increases [95,96], which agrees with discussion above that carcass weight becomes a greater percentage of the empty body as weight increases. Thus, the distribution of fat between carcass and offal is changing indicating a more sophisticated prediction is necessary. McPhee et al. [27] developed a model to estimate empty body fat distribution among four fat depots—intermuscular, intramuscular, subcutaneous, and visceral—with model mean biases of 0.34, −13.81, 17.75, and 16.56%, respectively. Development of such models could potentially improve prediction of empty body fat, particularly if carcass fat is already known.

Changes in proportion of offal in the empty body as empty body weight increases is likely the reason for the underprediction of empty body water and overprediction of empty body fat and energy at heavier empty body weights. Additionally, changes in the proportion of empty body water between carcass and offal due to genetic selection for increased muscling and/or management practices resulted in publication year impacting prediction of empty body water, which is supported by the opposite effect of publication year on fat-free dry matter. As discussed previously, carcass weight becomes a greater proportion of empty body weight, and carcass fat becomes a greater proportion of empty body fat as empty body weight increases.

Sex was not a factor in the analysis relating carcass chemical components to empty body chemical components as all studies used steers. Breed type had no effect on prediction of empty body chemical components except for ash. The overprediction of empty body ash in indicus compared with beef breed types is likely due to differences in carcass weight as a percentage of empty body weight as HCW of indicus breed types was overpredicted compared with beef breed types in the Weight dataset. In our dataset, carcass ash was a greater (*p* < 0.01) percentage of empty body ash (81.2 vs. 67.1 and 75.5%) in indicus than dairy and beef breed types, respectively.

Overall, the magnitude of the errors for predicting empty body chemical components were small (MB < 5%) with exception of fat for GH1969. Although offal (−3.81 to 3.22%) and EBW (−5.67 to 3.22%) affected prediction of empty body water, and offal (−3.78 to 5.02%) impacted prediction of empty body protein the magnitude of the errors were small to moderate. However, the magnitude of the errors associated with offal to predict empty body fat (−7.34 to 14.38%) and ash (−15.22 to 12.18%) were large. Additionally, the magnitude of the errors associated with EBW for prediction of empty body fat (−7.14 to 17.19%) were large.

### 4.4. Implications and Future Research

As the beef cattle industry looks to improve efficiency and sustainability, continued improvement in cattle nutrition will be necessary. Equations to predict body weight and chemical composition are used to determine nutrient requirements of cattle from nondestructive methods (i.e., carcass specific gravity, urea space, etc.). The inaccuracy of these equations negatively impacts estimates of nutrient requirements and predictions of feed intake and rate of gain, as these are based on data collected using these equations. Factors such as sex, breed type, genetic potential, and/or management system should be considered to improve prediction equations. Equations should be more robust across the full weight range of cattle production (birth to harvest) and likely need to be re-evaluated periodically as cattle genetics and production systems change.

One particularly concerning result of this analysis was the very poor prediction of empty body and carcass protein suggesting that estimates of metabolizable protein requirements may be inaccurate and imprecise for different phases of growth. This is supported by the poor coefficient of determination (R^2^ = 0.34) relating protein accretion with retained energy (i.e., fat accretion) [17]. Given that empty body protein was adequately predicted from known carcass protein, prediction of empty body protein may be improved by including anatomical measurements on the carcass. For example, separable lean of the 9–11th rib section is strongly related to protein of the carcass [97], thus measurements of longissimus muscle area may improve the prediction of carcass and empty body protein.

For 5 decades the constant of 0.077 Mcal/kg^.75^ has been used to estimate net energy for maintenance requirements for all classes of cattle with some crude adjustment factors for breed type [14,15,16,17], but maintenance energy requirements are also affected by sex, age, season, ambient temperature, physiological state, and previous nutrition [17]. Chemical protein composition is likely a primary driver of variation in maintenance energy requirements [11,98] in the form of visceral organs [7,8] but also skeletal muscle [4]. Chemical water, fat, and ash are metabolically inert substances being acted upon by enzymes, thus chemical protein is likely more strongly associated with net energy for maintenance requirements. In support of this hypothesis, chemical protein is associated with a greater maintenance energy requirement than chemical fat (188 vs. 20 kcal/kg, respectively) [9,10,11,12,13]. Thus, improved predictions of chemical protein composition could be used to increase our understanding of maintenance energy requirements leading to improved estimates of maintenance energy requirements according to sex, age, season, ambient temperature, physiological state, and previous nutrition.

## 5. Conclusions

Existing equations to predict body weight were highly precise, but were somewhat lacking in accuracy. Additionally, equations were generally adequate to predict fat concentration from known water concentration, but subsequent prediction of body composition was woefully inadequate. To better predict body chemical composition, a better understanding of the relationship of fat-free dry matter with protein and ash is needed. Prediction of chemical components was impacted by body weight and offal chemical composition, sometimes with considerable magnitude, indicating that current equations may not accurately adjust for changes in proportion of chemical components in the carcass and offal. Current equations were also frequently affected by sex and breed type indicating that adjustments should be made for these factors. Overall, the significant effect of publication year on prediction of weight, fat, protein, and ash concentrations indicates the compositional relationships among chemical components of the body have changed due to genetics and/or management of growing/finishing cattle, and that relationships need to be re-evaluated in current beef cattle production systems.

## Figures and Tables

**Table 1:** Summary statistics of body weight and chemical composition of cattle in the Weight dataset.

Item ^1^	N	Mean	SD	Min	Max
Shrunk body wt., kg	359	372.8	144.5	32.6	787.7
Empty body wt., kg	388	338.2	135.3	31.2	729.4
Hot carcass wt., kg	352	221.3	95.4	18.2	539.6
EB fat, % EBW	254	18.20	7.62	2.00	34.92
EB protein, % EBW	238	18.00	2.08	13.47	24.06

^1^ EB, empty body; EBW, empty body weight.

**Table 2:** Model evaluation of equations in predicting empty body weight computed from shrunk body weight, and predictive variables of the deviance between observed and predicted empty body weight using the Weight dataset.

Item ^1^	Gil1970	NRC2016
SRC β_0_	−10.65 ± 2.56 *	−18.41 ± 2.61 *
SRC β_1_	1.00 ± 0.01	1.05 ± 0.01 *
r^2^	0.9830	0.9830
Pr > F	<0.0001	<0.0001
RMSE	17.85	17.85
RMSEP	20.87	19.12
CCC	0.988	0.989
Cb	0.997	0.998
MB	−10.89	−0.38
% MB	−3.22	−0.11
CD	1.009	1.130
Deviance Predictors ^2^		
F values		
Year	0.21	4.39
Sex	39.97	52.79
Breed	11.26	24.05
R^2^	0.2685	0.3249
Year MRC β_1_	−0.038 ± 0.082	0.178 ± 0.085 *
Year means		
1970	−12.90	−2.98
2020	−12.10	5.95
Sex means		
Bull	5.11 ^b^	17.46 ^b^
Heifer	−14.21 ^a^	−6.01 ^a^
Steer	−15.69 ^a^	−7.17 ^a^
Breed means		
Dairy	−14.15 ^a^	−6.04 ^a^
Indicus	−5.36 ^b^	2.84 ^b^
Beef	−5.28 ^b^	7.47 ^b^

^1^ Gil1970, equation of Gil et al. [33]; NRC2016, equation of NASEM [17]; SRC, simple linear regression coefficient; r^2^, simple coefficient of determination; Pr > F, *p*-value for simultaneous test of intercept = 0 and slope = 1; RMSE = root mean square error; RMSEP = root mean square error of prediction; CCC = concordance correlation coefficient; Cb = bias correction factor; MB = mean bias; CD = coefficient of model determination. ^2^ R^2^, multiple coefficient of determination; Year, publication year; MRC, multiple linear regression coefficient. * Regression coefficient is significantly different from zero or one at *p* < 0.05. ^ab^ Means without a common superscript differ at *p* < 0.05.

**Table 3:** Model evaluation of equations predicting carcass weight computed from empty body weight, and predictive variables of the deviance between observed and predicted carcass weight in the Weight dataset.

Item ^1^	Lofgreen1962	GH1969	HL1969_1	HL1969_2	Gil1970	Alhassan1975	Ferrell1976	Fox1976
SRC β_0_	9.66 ± 1.46 *	8.61 ± 1.47 *	16.14 ± 1.43 *	29.48 ± 1.35 *	−1.50 ± 1.53	24.23 ± 1.38 *	4.39 ± 1.49 *	15.45 ± 1.43 *
SRC β_1_	1.00 ± 0.01	0.94 ± 0.01 *	0.99 ± 0.01	0.96 ± 0.01 *	1.03 ± 0.01 *	0.86 ± 0.01 *	0.97 ± 0.01 *	0.96 ± 0.01 *
r^2^	0.9862	0.9862	0.9862	0.9862	0.9862	0.9862	0.9862	0.9862
Pr > F	<0.0001	<0.0001	<0.0001	<0.0001	<0.0001	<0.0001	<0.0001	<0.0001
RMSE	11.21	11.21	11.21	11.21	11.21	11.21	11.21	11.21
RMSEP	13.74	13.41	17.22	22.77	8.866	19.89	11.10	13.30
CCC	0.988	0.990	0.982	0.970	0.992	0.979	0.992	0.989
Cb	0.995	0.996	0.989	0.977	0.998	0.986	0.999	0.996
MB	9.110	−5.716	14.178	20.684	4.286	−8.258	−1.867	7.546
% MB	4.12	−2.58	6.41	9.35	1.94	−3.73	−0.84	3.41
CD	0.936	0.834	0.911	0.829	1.000	0.698	0.899	0.876
Deviance Predictors ^2^								
F values								
Year	25.82	5.36	22.94	10.23	37.26	0.15	15.56	12.41
Sex	6.58	11.60	7.31	10.45	3.60	12.57	9.15	9.93
Breed	10.67	3.53	9.03	4.01	18.96	9.61	5.62	4.56
R^2^	0.2016	0.1338	0.1920	0.1494	0.2395	0.1257	0.1672	0.1566
Year MRC β_1_	0.256 ± 0.050 *	0.139 ± 0.060 *	0.243 ± 0.051 *	0.178 ± 0.055 *	0.307 ± 0.050 *	−0.035 ± 0.090	0.209 ± 0.053 *	0.192 ± 0.054 *
Year means								
1970	1.09	−9.92	6.57	15.20	−5.42	−6.72	−8.34	1.63
2020	13.87	−2.94	18.73	24.10	9.92	−8.47	2.09	11.21
Sex means								
Bull	2.92 ^a^	−13.36 ^a^	7.83 ^a^	13.50 ^a^	−1.26 ^a^	−18.09 ^a^	−8.65 ^a^	0.55 ^a^
Heifer	10.46 ^ab^	−1.42 ^b^	15.85 ^ab^	24.00 ^b^	4.34 ^ab^	0.46 ^b^	0.68 ^b^	10.53 ^b^
Steer	8.17 ^b^	−4.99 ^b^	13.42 ^b^	20.90 ^b^	2.62 ^b^	−5.03 ^b^	−2.13 ^b^	7.53 ^b^
Breed means								
Dairy	7.71 ^b^	−3.70 ^a^	13.14 ^b^	21.60 ^b^	1.38 ^a^	−1.10 ^b^	−1.88 ^b^	8.03 ^b^
Indicus	2.76 ^a^	−10.52 ^b^	7.99 ^a^	15.40 ^a^	−2.75 ^a^	−10.70 ^a^	−7.59 ^a^	2.05 ^a^
Beef	11.09 ^c^	−5.55 ^ab^	15.96 ^b^	21.50 ^b^	7.07 ^b^	−10.80 ^a^	−0.62 ^b^	8.52 ^b^

^1^ Lofgreen1962, equation of Lofgreen et al. [31]; GH1969, equation of Garrett and Hinman [21]; HL1969_1, Equation 1 of Holzer and Levy [32]; HL1969_2, Equation 2 of Holzer and Levy [32]; Gil1970, equation of Gil et al. [33]; Alhassan1975, equation of Alhassan et al. [34]; Ferrell1976, equation of Ferrell et al. [22]; Fox1976, equation of Fox et al. [35]; RC, simple linear regression coefficient; r^2^, simple coefficient of determination; Pr > F, *p*-value for simultaneous test of intercept = 0 and slope = 1; RMSE = root mean square error; RMSEP = root mean square error of prediction; CCC = concordance correlation coefficient; Cb = bias correction factor; MB = mean bias; CD = coefficient of model determination. ^2^ R^2^, multiple coefficient of determination; Year, publication year; MRC, multiple linear regression coefficient. * Regression coefficient is significantly different from zero or one at *p* < 0.05. ^abc^ Means without a common superscript differ at *p* < 0.05.

**Table 4:** Summary statistics of empty body chemical composition of cattle in the Empty Body dataset.

Item ^1^	N	Mean	SD	Min	Max
Empty body wt., kg	218	304.2	127.4	31.2	729.4
EB water, % EBW	223	59.49	6.41	45.50	73.40
EB fat, % EBW	223	18.03	7.82	2.00	35.00
EB FFDM, % EBW	223	22.48	2.22	15.48	30.40
EB protein, % EBW	223	18.16	1.95	13.85	24.06
EB ash, % EBW	223	4.33	1.11	1.45	6.64
EB protein, % FFDM	223	80.83	4.66	70.00	94.32
EB ash, % FFDM	223	19.23	4.60	6.42	30.25

^1^ EB, empty body; EBW, empty body weight; FFDM, fat free dry matter.

**Table 5:** Model evaluation of predicting empty body chemical fat and fat-free dry matter assuming empty body water is known, and predictive variables of the deviance between observed and predicted empty body chemical components in the Empty Body dataset.

	Fat			Fat-Free Dry Matter		
Item ^1^	GH1969	Gil1970	Ferrell1976	GH1969	Gil1970	Ferrell1976
SRC β_0_	0.23 ± 0.32	−1.07 ± 0.34 *	0.97 ± 0.31 *	7.46 ± 1.59 *	12.14 ± 0.55 *	9.03 ± 1.43 *
SRC β_1_	0.94 ± 0.02 *	0.88 ± 0.01 *	0.91 ± 0.02 *	0.70 ± 0.07 *	0.55 ± 0.06 *	0.62 ± 0.07 *
r^2^	0.9429	0.9429	0.9429	0.2887	0.2887	0.2887
Pr > F	<0.0001	<0.0001	<0.0001	<0.0001	<0.0001	<0.0001
RMSE	1.873	1.873	1.873	1.873	1.873	1.873
RMSEP	2.167	4.135	2.08	2.167	4.135	2.12
CCC	0.963	0.884	0.966	0.462	0.232	0.504
Cb	0.992	0.910	0.994	0.861	0.432	0.939
MB	−0.978	−3.556	−0.686	0.978	3.556	0.686
% MB	−5.43	−19.73	−3.81	4.35	15.82	3.05
CD	0.917	0.708	0.875	1.267	0.281	1.171
Deviance Predictors ^2^						
F values						
EBW	0.34	11.61	3.80	0.54	11.61	3.80
Year	60.67	53.22	57.18	60.67	53.22	57.18
Sex	0.50	1.26	0.82	0.50	1.26	0.82
Breed	6.70	6.20	6.46	6.70	6.20	6.46
R^2^	0.2712	0.3364	0.2979	0.2712	0.3364	0.2979
EBW MRC β_1_	−0.0006 ± 0.001	−0.004 ± 0.001 *	−0.002 ± 0.001 *	0.0006 ± 0.001	0.004 ± 0.001 *	0.002 ± 0.001 *
EBW means						
35	−0.30	−1.96	0.43	0.30	1.96	−0.43
725	−0.73	−4.61	−1.05	0.73	4.61	1.05
Year MRC β_1_	−0.090 ± 0.01 *	−0.088 ± 0.01 *	−0.089 ± 0.01 *	0.090 ± 0.01 *	0.088 ± 0.01 *	0.089 ± 0.01 *
Year means						
1976	1.19	−1.37	1.49	−1.29	1.37	−1.49
2020	−2.77	−5.24	−2.43	2.77	5.24	2.43
Sex means						
Bulls	−0.08	−2.32	0.37	0.08	2.32	−0.37
Heifers	−0.83	−3.57	−0.62	0.83	3.57	0.62
Steers	−0.50	−3.08	−0.21	0.50	3.08	0.21
Breed means						
Dairy	−0.93 ^a^	−3.38 ^a^	−0.58 ^a^	0.93 ^b^	3.38 ^b^	0.58 ^b^
Indicus	0.72 ^b^	−1.85 ^b^	1.02 ^b^	−0.72 ^a^	1.85 ^a^	−1.02 ^a^
Beef	−1.20 ^a^	−3.75 ^a^	−0.89 ^a^	1.20 ^b^	3.75 ^b^	0.89 ^b^

^1^ GH1969, equation of Garrett and Hinman [21]; Gil1970, equation of Gil et al. [33]; Ferrell1976, equation of Ferrell et al. [22]; SRC, simple linear regression coefficient; r^2^, simple coefficient of determination; Pr > F, *p*-value for simultaneous test of intercept = 0 and slope = 1; RMSE = root mean square error; RMSEP = root mean square error of prediction; CCC = concordance correlation coefficient; Cb = bias correction factor; MB = mean bias; CD = coefficient of model determination. ^2^ EBW, empty body weight; Year, publication year; R^2^, multiple coefficient of determination; MRC, multiple linear regression coefficient. * Regression coefficient is significantly different from zero or one at *p* < 0.05. ^ab^ Means without a common superscript differ at *p* < 0.05.

**Table 6:** Model evaluation of predicting empty body chemical protein and ash assuming empty body water is known, and predictive variables of the deviance between observed and predicted empty body chemical components in the Empty Body dataset.

	Protein			Ash		
Item ^1^	GH1969	Gil1970	Ferrell1976	GH1969	Gil1970	Ferrell1976
SRC β_0_	5.75 ± 1.45 *	6.58 ± 1.36 *	6.46 ± 1.37 *	2.11 ± 0.88 *	3.57 ± 0.31 *	2.65 ± 0.67 *
SRC β_1_	0.69 ± 0.08 *	0.76 ± 0.09 *	0.67 ± 0.08 *	0.61 ± 0.24	0.21 ± 0.08 *	0.39 ± 0.15 *
r^2^	0.2485	0.2485	0.2485	0.0280	0.0280	0.0280
Pr > F	<0.0001	<0.0001	<0.0001	<0.0001	<0.0001	0.0005
RMSE	1.693	1.693	1.693	1.100	1.100	1.100
RMSEP	1.765	3.359	1.887	1.29	1.468	1.133
CCC	0.465	0.180	0.441	0.063	0.134	0.121
Cb	0.932	0.361	0.884	0.378	0.799	0.726
MB	0.303	2.889	0.701	0.682	0.674	−0.007
% MB	1.67	15.91	3.86	15.75	15.56	−0.16
CD	1.854	0.379	1.464	2.21	0.981	5.397
Deviance Predictors ^2^						
F values						
EBW	0.62	0.00	1.27	0.01	34.64	3.67
Year	19.72	20.33	19.45	38.14	27.66	35.06
Sex	0.17	0.23	0.16	3.13	6.10	3.97
Breed	16.64	16.69	16.61	7.97	5.83	7.29
R^2^	0.2190	0.2027	0.2274	0.2447	0.3513	0.2544
EBW MRC β_1_	0.0008 ± 0.001	0.0000 ± 0.001	0.0011 ± 0.001	0.00005 ± 0.001	0.0040 ± 0.001 *	0.0012 ± 0.001 †
EBW means						
35	−0.49	2.32	−0.19	0.72	−0.44	−0.31
725	0.06	2.32	0.59	0.75	2.36	0.54
Year MRC β_1_	0.048 ± 0.01 *	0.048 ± 0.01 *	0.047 ± 0.01 *	0.041 ± 0.01 *	0.039 ± 0.01 *	0.041 ± 0.01 *
Year means						
1976	−1.16	1.43	−0.76	−0.03	−0.06	−0.73
2020	0.95	3.56	1.33	1.79	1.64	1.06
Sex means						
Bulls	−0.18	2.49	0.18	0.15 ^a^	−0.28 ^a^	−0.66 ^a^
Heifers	−0.24	2.31	0.18	1.02 ^ab^	1.21 ^b^	0.39 ^b^
Steers	−0.42	2.16	−0.02	1.02 ^b^	1.02 ^b^	0.33 ^b^
Breed means						
Dairy	0.16 ^b^	2.78 ^b^	0.54 ^b^	0.65 ^b^	0.48 ^ab^	−0.08 ^ab^
Indicus	−1.85 ^a^	0.73 ^a^	−1.46 ^a^	1.30 ^b^	1.28 ^b^	0.61 ^b^
Beef	0.86 ^c^	3.45 ^c^	1.25 ^c^	0.23 ^a^	0.19 ^a^	−0.47 ^a^

^1^ GH1969, equation of Garrett and Hinman [21]; Gil1970, equation of Gil et al. [33]; Ferrell1976, equation of Ferrell et al. [22]; SRC, simple linear regression coefficient; r^2^, simple coefficient of determination; Pr > F, *p*-value for simultaneous test of intercept = 0 and slope = 1; RMSE = root mean square error; RMSEP = root mean square error of prediction; CCC = concordance correlation coefficient; Cb = bias correction factor; MB = mean bias; CD = coefficient of model determination. ^2^ EBW, empty body weight; Year, publication year; R^2^, multiple coefficient of determination; MRC, multiple linear regression coefficient. * Regression coefficient is significantly different from zero or one at *p* < 0.05. † Regression coefficient is significantly different from zero at *p* < 0.10. ^abc^ Means without a common superscript differ at *p* < 0.05.

**Table 7:** Model evaluation of predicting empty body energy assuming empty body water is known, and predictive variables of the deviance between observed and predicted empty body chemical components in the Empty Body dataset.

	Energy1		Energy2		
Item ^1^	GH1969	Ferrell1976	GH1969	Gil1970	Ferrell1976
SRC β_0_	0.02 ± 0.02	0.09 ± 0.02 *	0.02 ± 0.03	0.12 ± 0.03 *	0.14 ± 0.03 *
SRC β_1_	1.00 ± 0.01	0.99 ± 0.01	0.96 ± 0.01 *	0.90 ± 0.01 *	0.94 ± 0.01 *
r^2^	0.9850	0.9849	0.9709	0.9709	0.9709
Pr > F	<0.0001	<0.0001	<0.0001	<0.0001	<0.0001
RMSE	0.082	0.083	0.115	0.115	0.116
RMSEP	0.086	0.103	0.138	0.222	0.125
CCC	0.991	0.988	0.979	0.952	0.984
Cb	0.999	0.996	0.994	0.966	0.998
MB	0.028	0.061	−0.075	−0.174	−0.026
% MB	1.03	2.23	−2.78	−6.42	−0.94
CD	1.017	0.987	0.946	0.785	0.907
Deviance Predictors ^2^					
F values					
EBW	0.11	0.69	0.05	25.66	4.25
Year	12.82	14.33	69.04	53.54	63.41
Sex	0.32	0.47	0.21	3.42	2.07
Breed	17.70	17.58	1.38	0.36	0.63
R^2^	0.1961	0.1794	0.2683	0.3702	0.2923
EBW MRC β_1_	0.00002 ± 0.0001	0.0004 ± 0.0001	0.00001 ± 0.0001	−0.0003 ± 0.0001 *	−0.0001 ± 0.00001 *
EBW means					
35	−0.004	0.04	−0.05	−0.06	0.03
725	0.007	0.02	−0.06	−0.31	−0.07
Year MRC β_1_	0.0018 ± 0.001 *	0.0020 ± 0.001 *	−0.0058 ± 0.001 *	−0.0056 ± 0.001 *	−0.0057 ± 0.001 *
Year means					
1976	−0.03	0.00	0.05	−0.05	0.10
2020	0.05	0.08	−0.21	−0.29	−0.15
Sex means					
Bulls	0.011	0.05	−0.02	−0.08 ^b^	0.04
Heifers	−0.002	0.03	−0.09	−0.21 ^a^	−0.05
Steers	−0.008	0.02	−0.07	−0.17 ^a^	−0.02
Breed means					
Dairy	0.02 ^b^	0.06 ^b^	−0.08	−0.16	−0.02
Indicus	−0.08 ^a^	−0.05 ^a^	−0.04	−0.13	0.01
Beef	0.06 ^c^	0.09 ^c^	−0.06	−0.16	−0.01

^1^ GH1969, equation of Garrett and Hinman [21]; Gil1970, equation of Gil et al. [33]; Ferrell1976, equation of Ferrell et al. [22]; Energy1, energy concentration predicted from observed fat percentage; Energy2, energy concentration calculated from predicted fat and protein percentages; SRC, simple linear regression coefficient; r^2^, simple coefficient of determination; Pr > F, *p*-value for simultaneous test of intercept = 0 and slope = 1; RMSE = root mean square error; RMSEP = root mean square error of prediction; CCC = concordance correlation coefficient; Cb = bias correction factor; MB = mean bias; CD = coefficient of model determination. ^2^ EBW, empty body weight; Year, publication year; R^2^, multiple coefficient of determination; MRC, multiple linear regression coefficient. * Regression coefficient is significantly different from zero or one at *p* < 0.05. ^abc^ Means without a common superscript differ at *p* < 0.05.

**Table 8:** Summary statistics of carcass chemical composition of cattle in the Carcass dataset.

Item ^1^	N	Mean	SD	Min	Max
Carcass wt., kg	122	227.7	98.2	18.2	539.6
Water, % HCW	127	56.35	7.67	37.78	74.30
Fat, % HCW	127	21.07	9.10	1.80	40.75
FFDM, % HCW	127	22.57	2.97	14.96	30.49
Protein, % HCW	127	17.62	2.82	12.66	24.38
Ash, % HCW	127	4.80	1.74	1.45	8.17
Protein, % FFDM	127	78.18	8.16	58.46	93.75
Ash, % FFDM	127	21.18	7.22	6.25	37.25

^1^ HCW, hot carcass weight; FFDM, fat free dry matter.

**Table 9:** Model evaluation of predicting carcass chemical fat and fat-free dry matter assuming carcass water is known, and predictive variables of the deviance between observed and predicted carcass chemical components in the Carcass dataset.

	Fat				Fat-Free Dry Matter			
Item ^1^	GH1969	Gil1970	Preston1974	Ferrell1976	GH1969	Gil1970	Preston1974	Ferrell1976
SRC β_0_	2.14 ± 0.60 *	1.48 ± 0.62 *	2.41 ± 0.59 *	1.88 ± 0.61 *	14.45 ± 2.07 *	15.12 ± 1.90 *	13.32 ± 2.36 *	14.29 ± 2.11 *
SRC β_1_	0.84 ± 0.02 *	0.84 ± 0.02 *	0.86 ± 0.02 *	0.85 ± 0.02 *	0.38 ± 0.10 *	0.37 ± 0.09 *	0.42 ± 0.11 *	0.39 ± 0.10 *
r^2^	0.9052	0.9052	0.9052	0.9052	0.1109	0.1109	0.1109	0.1109
Pr > F	<0.0001	<0.0001	<0.0001	<0.0001	<0.0001	<0.0001	<0.0001	<0.0001
RMSE	2.812	2.812	2.812	2.812	2.812	2.812	2.812	2.812
RMSEP	3.481	4.026	3.149	3.514	3.481	4.026	3.149	3.514
CCC	0.936	0.916	0.945	0.934	0.295	0.245	0.318	0.286
Cb	0.983	0.963	0.994	0.982	0.886	0.737	0.955	0.858
MB	−1.343	−2.361	−0.531	−1.500	1.343	2.361	0.531	0.1.500
% MB	−6.37	−11.20	−2.52	−7.12	5.95	10.46	2.35	6.65
CD	0.774	0.804	0.822	0.781	1.038	0.688	1.512	1.026
Deviance Predictors ^2^								
F values								
HCW	12.59	15.70	6.60	10.61	12.59	15.70	6.60	10.61
Year	101.15	98.61	107.06	102.93	101.15	98.61	107.06	102.93
Sex	1.95	2.08	1.66	1.86	1.95	2.08	1.66	1.86
Breed	3.37	3.26	3.63	3.45	3.37	3.26	3.63	3.45
R^2^	0.6050	0.6108	0.5920	0.6010	0.6050	0.6108	0.5920	0.6010
HCW MRC β_1_	−0.007 ± 0.002 *	−0.009 ± 0.002 *	−0.005 ± 0.002 *	−0.007 ± 0.002 *	0.008 ± 0.002 *	0.0087 ± 0.002 *	0.0055 ± 0.002 *	0.007 ± 0.002 *
HCW means								
20	1.39	0.59	1.68	1.07	−1.39	−0.59	−1.68	−1.07
535	−2.59	−3.89	−1.15	−2.56	2.59	3.89	1.15	2.56
Year MRC β_1_	−0.178 ± 0.02 *	−0.177 ± 0.02 *	−0.180 ± 0.02 *	−0.179 ± 0.02 *	0.178 ± 0.02 *	0.177 ± 0.02 *	0.180 ± 0.02 *	0.179 ± 0.02 *
Year means								
1976	3.33	2.32	4.13	3.17	−3.33	−2.32	−4.13	−3.17
2020	−4.51	−5.48	−3.80	−4.70	4.51	5.48	3.80	4.70
Sex means								
Bulls	0.52	−0.44	1.21	0.32	−0.52	0.44	−1.21	−0.32
Steers	−0.96	−1.98	−0.13	−1.11	0.96	1.98	0.13	1.11
Breed means								
Dairy	−0.48 ^ab^	−1.46 ^ab^	0.26 ^ab^	−0.65 ^ab^	0.48 ^ab^	1.46 ^ab^	−0.26 ^ab^	0.65 ^ab^
Indicus	0.85 ^b^	−0.15 ^b^	1.64 ^b^	0.69 ^b^	−0.85 ^a^	0.15 ^a^	−1.64 ^a^	−0.69 ^b^
Beef	−1.03 ^a^	−2.02 ^a^	−0.29 ^a^	−1.21 ^a^	1.03 ^b^	2.02 ^b^	0.29 ^b^	1.21 ^a^

^1^ GH1969, equation of Garrett and Hinman [21]; Gil1970, equation of Gil et al. [33]; Preston1974, equation of Preston et al. [36]; Ferrell1976, equation of Ferrell et al. [22]; SRC, simple linear regression coefficient; r^2^, simple coefficient of determination; Pr > F, *p*-value for simultaneous test of intercept = 0 and slope = 1; RMSE = root mean square error; RMSEP = root mean square error of prediction; CCC = concordance correlation coefficient; Cb = bias correction factor; MB = mean bias; CD = coefficient of model determination. ^2^ HCW, hot carcass weight; Year, publication year; R^2^, multiple coefficient of determination; MRC, multiple linear regression coefficient. * Regression coefficient is significantly different from zero or one at *p* < 0.05. ^ab^ Means without a common superscript differ at *p* < 0.05.

**Table 10:** Model evaluation of predicting carcass chemical protein and ash assuming carcass water is known, and predictive variables of the deviance between observed and predicted carcass chemical components in the Carcass dataset.

	Protein				Ash			
Item ^1^	GH1969	Gil1970	Preston1974	Ferrell1976	GH1969	Gil1970	Preston1974	Ferrell1976
SRC β_0_	7.45 ± 1.85 *	5.49 ± 2.21 *	6.45 ± 2.03 *	7.06 ± 1.92 *	5.21 ± 1.33 *	4.99 ± 0.62 *	5.34 ± 1.74 *	5.19 ± 1.27 *
SRC β_1_	0.61 ± 0.11 *	0.74 ± 0.13	0.65 ± 0.12 *	0.66 ± 0.12 *	−0.09 ± 0.29 *	−0.05 ± 0.15 *	−0.11 ± 0.35 *	−0.08 ± 0.25 *
r^2^	0.1961	0.1961	0.1961	0.1961	0.0008	0.0008	0.0008	0.0008
Pr > F	<0.0001	<0.0001	0.0018	<0.0001	0.0003	<0.0001	0.0072	0.0003
RMSE	2.539	2.539	2.539	2.539	1.741	1.741	1.741	1.741
RMSEP	2.791	2.842	2.649	3.038	1.845	2.236	1.797	1.864
CCC	0.395	0.341	0.404	0.337	−0.015	−0.019	−0.013	−0.017
Cb	0.892	0.770	0.912	0.762	0.546	0.699	0.474	0.622
MB	0.897	1.241	0.478	1.571	0.293	0.966	−0.101	−0.224
% MB	5.09	7.04	2.72	8.92	6.09	20.12	−2.10	−4.66
CD	1.582	1.808	2.036	1.306	8.167	1.556	14.560	6.976
Deviance Predictors ^2^								
F values								
HCW	2.86	0.01	1.23	1.06	2.80	20.19	1.16	4.91
Year	23.00	24.94	23.72	23.83	32.44	26.41	33.70	31.29
Sex	2.17	2.84	2.41	2.44	14.68	16.18	14.33	14.99
Breed	14.87	15.53	15.13	15.16	12.36	11.95	12.41	12.30
R^2^	0.3662	0.3327	0.3520	0.3501	0.4204	0.4815	0.4118	0.4298
HCW MRC β_1_	0.004 ± 0.002	0.0003 ± 0.002	0.0025 ± 0.002	0.0023 ± 0.002	0.0025 ± 0.001	0.007 ± 0.001 *	−0.0016 ± 0.001	0.0033 ± 0.002 *
HCW means								
20	−0.26	0.91	−0.37	0.76	−0.89	−1.28	−1.08	−1.61
535	1.72	1.05	0.92	1.96	0.39	2.35	−0.26	0.11
Year MRC β_1_	0.088 ± 0.02 *	0.091 ± 0.02 *	0.090 ± 0.02 *	0.090 ± 0.02 *	0.069 ± 0.01 *	0.065 ± 0.01 *	0.070 ± 0.01 *	0.068 ± 0.01 *
Year means								
1976	−1.23	−0.86	−1.64	−0.55	−1.76	−1.12	−2.14	−2.28
2020	2.67	3.17	2.31	3.41	1.29	1.77	0.94	0.74
Sex means								
Bulls	1.35	1.89	1.00	2.10	−1.77 ^a^	−1.34 ^a^	−2.11 ^a^	−2.33 ^a^
Steers	−0.28	0.04	0.70	0.39	1.01 ^b^	1.72 ^b^	0.62 ^b^	0.50 ^b^
Breed means								
Dairy	1.20 ^b^	1.66 ^b^	0.82 ^a^	1.92 ^b^	−1.10 ^a^	−0.57 ^a^	−1.46 ^a^	−1.64 ^a^
Indicus	−1.88 ^a^	−1.49 ^a^	−2.28 ^b^	−1.19 ^a^	1.25 ^b^	1.87 ^b^	0.87 ^b^	0.72 ^b^
Beef	2.28 ^b^	2.73 ^b^	1.90 ^a^	3.00 ^b^	−1.28 ^a^	−0.74 ^a^	−1.64 ^a^	−1.82 ^a^

^1^ GH1969, equation of Garrett and Hinman [21]; Gil1970, equation of Gil et al. [33]; Preston1974, equation of Preston et al. [36]; Ferrell1976, equation of Ferrell et al. [22]; SRC, simple linear regression coefficient; r^2^, simple coefficient of determination; Pr > F, *p*-value for simultaneous test of intercept = 0 and slope = 1; RMSE = root mean square error; RMSEP = root mean square error of prediction; CCC = concordance correlation coefficient; Cb = bias correction factor; MB = mean bias; CD = coefficient of model determination. ^2^ HCW, hot carcass weight; Year, publication year; R^2^, multiple coefficient of determination; MRC, multiple linear regression coefficient. * Regression coefficient is significantly different from zero or one at *p* < 0.05. ^ab^ Means without a common superscript differ at *p* < 0.05.

**Table 11:** Model evaluation of predicting carcass energy content assuming carcass water is known, and predictive variables of the deviance between observed and predicted carcass chemical components in the Carcass dataset.

	Energy1		Energy2			
Item ^1^	GH1969	Ferrell1976	GH1969	Gil1970	Preston1974	Ferrell1976
SRC β_0_	0.03 ± 0.04	0.16 ± 0.04 *	0.30 ± 0.06 *	0.32 ± 0.06 *	0.30 ± 0.06 *	0.33± 0.06 *
SRC β_1_	1.00 ± 0.01	0.97 ± 0.01 *	0.88 ± 0.02 *	0.85 ± 0.02 *	0.89 ± 0.02 *	0.87 ± 0.02 *
r^2^	0.9746	0.9755	0.9408	0.9407	0.9407	0.9406
Pr > F	0.0043	<0.0001	<0.0001	<0.0001	<0.0001	0.0006
RMSE	0.123	0.122	0.187	0.188	0.188	0.189
RMSEP	0.127	0.147	0.226	0.277	0.209	0.224
CCC	0.986	0.982	0.961	0.944	0.966	0.962
Cb	0.999	0.994	0.990	0.974	0.996	0.992
MB	0.027	0.080	−0.076	−0.154	−0.024	−0.055
% MB	1.24	2.71	−2.58	−5.21	−0.80	−1.88
CD	1.030	0.955	0.809	0.739	0.842	0.808
Deviance Predictors ^2^						
F values						
HCW	1.07	1.70	12.44	27.98	6.99	13.44
Year	10.98	16.17	99.84	88.74	106.10	99.99
Sex	4.00	4.78	10.51	11.15	10.03	10.39
Breed	16.09	16.68	0.58	0.60	0.51	0.51
R^2^	0.2968	0.2807	0.6011	0.6261	0.5915	0.6045
HCW MRC β_1_	0.00011 ± 0.0001	0.0001 ± 0.0001	−0.00051 ± 0.0001 *	−0.0008 ± 0.0001 *	−0.0004 ± 0.0001 *	−0.0005 ± 0.0001 *
HCW means						
20	0.01	0.11	0.12	0.11	0.14	0.14
535	0.06	0.03	−0.15	−0.31	−0.06	−0.13
Year MRC β_1_	0.0030 ± 0.001 *	0.0037 ± 0.001 *	−0.0118 ± 0.001 *	−0.012 ± 0.001 *	−0.012 ± 0.001 *	−0.012 ± 0.001 *
Year means						
1976	−0.03	0.00	0.24	0.17	0.30	0.27
2020	0.10	0.16	−0.28	−0.34	−0.23	−0.26
Sex means						
Bulls	0.08 ^b^	0.14 ^b^	0.12 ^b^	0.06 ^b^	0.17 ^b^	0.15 ^b^
Steers	−0.02 ^a^	0.02 ^a^	−0.10 ^a^	−0.18 ^a^	−0.05 ^a^	−0.08 ^a^
Breed means						
Dairy	0.06 ^b^	0.11 ^b^	0.02	−0.05	0.07	0.04
Indicus	−0.09 ^a^	−0.05 ^a^	−0.02	−0.10	0.03	0.00
Beef	0.12 ^b^	0.17 ^b^	0.03	−0.04	0.08	0.05

^1^ GH1969, equation of Garrett and Hinman [21]; Gil1970, equation of Gil et al. [33]; Preston1974, equation of Preston et al. [36]; Ferrell1976, equation of Ferrell et al. [22]; Energy1, energy concentration predicted from observed fat percentage; Energy2, energy concentration calculated from predicted fat and protein percentages; SRC, simple linear regression coefficient; r^2^, simple coefficient of determination; Pr > F, *p*-value for simultaneous test of intercept = 0 and slope = 1; RMSE = root mean square error; RMSEP = root mean square error of prediction; CCC = concordance correlation coefficient; Cb = bias correction factor; MB = mean bias; CD = coefficient of model determination. ^2^ HCW, hot carcass weight; Year, publication year; R^2^, multiple coefficient of determination; MRC, multiple linear regression coefficient. * Regression coefficient is significantly different from zero or one at *p* < 0.05. ^ab^ Means without a common superscript differ at *p* < 0.05.

**Table 12:** Summary statistics of empty body and carcass chemical composition of cattle in the Empty Body-Carcass dataset.

Item ^1^	N	Mean	SD	Min	Max
Empty body wt., kg	96	327.1	145.7	31.2	729.4
EB water, % EBW	101	58.05	6.97	45.50	73.40
EB fat, % EBW	101	19.20	8.01	2.00	33.50
EB FFDM, % EBW	101	22.74	2.17	17.93	30.41
EB protein, % EBW	101	18.51	2.25	14.08	24.06
EB ash, % EBW	101	4.27	1.25	1.45	6.55
EB protein, % FFDM	101	81.32	5.34	70.63	93.58
EB ash, % FFDM	101	18.86	5.45	6.42	30.25
Carcass wt., kg	96	220.1	106.2	18.2	539.6
Carcass water, % HCW	101	56.81	8.12	37.78	74.30
Carcass fat, % HCW	101	20.14	9.15	1.80	40.75
Carcass FFDM, % HCW	101	23.05	2.64	17.89	30.49
Carcass protein, % HCW	101	17.87	2.97	12.66	24.38
Carcass ash, % HCW	101	5.01	1.70	1.45	8.17
Carcass protein, % FFDM	101	77.45	8.54	58.46	93.75
Carcass ash, % FFDM	101	21.88	7.53	6.25	37.25

^1^ EB, empty body; EBW, empty body weight; FFDM, fat free dry matter.

**Table 13:** Model evaluation of predicting empty body chemical water, fat, and fat-free dry matter assuming carcass chemical composition is known, and predictive variables of the deviance between observed and predicted empty body chemical components in the Empty Body-Carcass dataset.

	Water		Fat		Fat-Free Dry Matter	
Item ^1^	GH1969	Ferrell1976	GH1969	Ferrell1976	GH1969	Ferrell1976
SRC β_0_	6.95 ± 1.10 *	0.42 ± 1.24	2.51 ± 0.37 *	0.18 ± 0.41	3.52 ± 0.86 *	1.47 ± 0.77
SRC β_1_	0.86 ± 0.02 *	0.99 ± 0.02	0.93 ± 0.02 *	1.02 ± 0.02	0.84 ± 0.04 *	0.92 ± 0.03 *
r^2^	0.9566	0.9566	0.9617	0.9617	0.8371	0.8861
Pr > F	<0.0001	0.3666	<0.0001	0.0038	<0.0001	<0.0001
RMSE	1.461	1.461	1.575	1.575	0.880	0.736
RMSEP	2.039	1.461	2.072	1.650	0.985	0.816
CCC	0.962	0.977	0.968	0.978	0.906	0.931
Cb	0.984	0.999	0.987	0.997	0.990	0.989
MB	−0.977	−0.194	1.224	0.519	−0.247	−0.325
% MB	−1.68	−0.33	6.38	2.70	−1.09	−1.431
CD	0.771	1.023	0.878	1.073	0.826	0.939
Deviance Predictors ^2^						
F values						
Offal	1.32	11.98	1.85	8.08	14.53	33.14
EBW	8.08	5.74	5.26	3.20	0.40	3.02
Year	8.11	7.60	0.03	0.21	46.59	32.08
Breed	1.97	1.85	0.79	0.95	1.37	0.84
R^2^	0.4102	0.3057	0.1177	0.1521	0.6075	0.5464
Offal MRC β_1_	0.060 ± 0.05	0.157 ± 0.04 *	0.068 ± 0.05	0.130 ± 0.04 *	0.141 ± 0.04 *	0.179 ± 0.03 *
Offal means						
Min	−1.88	−2.21	0.27	−1.41	−0.58	−0.86
Max	−0.32	1.87	2.46	2.76	0.98	1.11
EBW MRC β_1_	0.007 ± 0.002 *	0.005 ± 0.002 *	−0.007 ± 0.003 *	−0.005 ± 0.003	−0.0004 ± 0.001	0.001 ± 0.001
EBW means						
35	−3.29	−1.86	3.30	2.00	0.01	−0.54
725	1.78	1.87	−1.37	−1.32	−0.27	0.10
Year MRC β_1_	0.047 ± 0.02 *	0.040 ± 0.01 *	0.003 ± 0.02	−0.008 ± 0.02	−0.048 ± 0.01 *	−0.033 ± 0.006 *
Year means						
1981	−1.91	−0.93	1.27	0.72	0.67	0.28
2020	−0.06	0.63	1.40	0.42	−1.20	−1.03
Breed means						
Dairy	−0.53	0.32	0.83	0.08	−0.11	−0.36
Indicus	−1.94	−0.98	1.91	1.18	0.08	−0.10
Beef	−0.96	−0.19	1.23	0.52	−0.29	−0.34

^1^ GH1969, equation of Garrett and Hinman [21]; Ferrell1976, equation of Ferrell et al. [22]; SRC, simple linear regression coefficient; r^2^, simple coefficient of determination; Pr > F, *p*-value for simultaneous test of intercept = 0 and slope = 1; RMSE = root mean square error; RMSEP = root mean square error of prediction; CCC = concordance correlation coefficient; Cb = bias correction factor; MB = mean bias; CD = coefficient of model determination. ^2^ Offal, head, hide, feet and ears, blood, internal organs and fat; EBW, empty body weight; Year, publication year; R^2^, multiple coefficient of determination; MRC, multiple linear regression coefficient. * Regression coefficient is significantly different from zero or one at *p* < 0.05.

**Table 14:** Model evaluation of predicting empty body chemical protein, ash, and energy assuming carcass chemical composition is known, and predictive variables of the deviance between observed and predicted empty body chemical components in the Empty Body-Carcass dataset.

	Protein		Ash		Energy	
Item ^1^	GH1969	Ferrell1976	GH1969	Ferrell1976	GH1969	Ferrell1976
SRC β_0_	1.09 ± 0.43 *	1.78 ± 0.41 *	−0.21 ± 0.10 *	0.55 ± 0.08 *	0.32 ± 0.05 *	−0.02 ± 0.06
SRC β_1_	0.95 ± 0.02 *	0.90 ± 0.02 *	1.04 ± 0.02	0.83 ± 0.02 *	0.92 ± 0.02 *	1.03 ± 0.02
r^2^	0.9445	0.9445	0.9583	0.9583	0.9636	0.9636
Pr > F	0.0017	<0.0001	0.0841	<0.0001	<0.0001	<0.0001
RMSE	0.534	0.534	0.257	0.257	0.132	0.133
RMSEP	0.563	0.591	0.260	0.402	0.187	0.147
CCC	0.969	0.968	0.977	0.957	0.965	0.976
Cb	0.997	0.996	0.998	0.977	0.983	0.995
MB	0.157	−0.101	−0.028	−0.194	0.122	0.062
% MB	0.85	−0.54	−0.65	−4.54	4.33	2.17
CD	0.949	0.854	1.135	1.135	0.863	1.089
Deviance Predictors ^2^						
F values						
Offal	17.64	6.39	541.70	42.43	4.69	14.19
EBW	2.38	2.21	0.27	0.46	10.72	5.81
Year	0.09	0.15	30.40	3.33	0.00	0.30
Breed	1.72	2.87	8.10	17.82	2.50	2.12
R^2^	0.2286	0.1443	0.8686	0.5347	0.2249	0.2559
Offal MRC β_1_	0.162 ± 0.04 *	0.112 ± 0.04 *	0.262 ± 0.01 *	0.189 ± 0.03 *	0.099 ± 0.04 *	0.156 ± 0.04 *
Offal means						
Min	−0.37	−0.44	−0.52	−0.65	0.01	−0.13
Max	0.93	0.46	0.52	0.11	0.29	0.30
EBW MRC β_1_	0.001 ± 0.000	0.001 ± 0.001	0.000 ± 0.000	0.000 ± 0.000	−0.001 ± 0.000 *	−0.001 ± 0.000 *
EBW means						
35	−0.05	−0.32	−0.09	−0.37	0.36	0.22
725	0.52	0.31	−0.05	−0.26	−0.16	−0.13
Year MRC β_1_	0.002 ± 0.005	−0.002 ± 0.006	0.006 ± 0.001 *	−0.005 ± 0.003	0.000 ± 0.001	−0.001 ± 0.001
Year means						
1981	0.17	−0.01	−0.18	−0.23	0.14	0.08
2020	0.23	−0.10	0.07	−0.44	0.14	0.06
Breed means						
Dairy	−0.03	−0.35	−0.05 ^ab^	−0.12 ^a^	0.08	0.01
Indicus	0.44	0.31	−0.16 ^a^	−0.68 ^b^	0.22	0.14
Beef	0.16	−0.10	−0.02 ^b^	−0.15 ^a^	0.12	0.06

^1^ GH1969, equation of Garrett and Hinman [21]; Ferrell1976, equation of Ferrell et al. [22]; SRC, simple linear regression coefficient; r^2^, simple coefficient of determination; Pr > F, *p*-value for simultaneous test of intercept = 0 and slope = 1; RMSE = root mean square error; RMSEP = root mean square error of prediction; CCC = concordance correlation coefficient; Cb = bias correction factor; MB = mean bias; CD = coefficient of model determination. ^2^ Offal, head, hide, feet and ears, blood, internal organs and fat; EBW, empty body weight; Year, publication year; R^2^, multiple coefficient of determination; MRC, multiple linear regression coefficient. * Regression coefficient is significantly different from zero or one at *p* < 0.05. ^ab^ Means without a common superscript differ at *p* < 0.05.

## Data Availability

Data are available upon request.

## Supplementary Materials

The following can be downloaded at: https://www.mdpi.com/article/10.3390/ani12243554/s1, Table S1. Equations evaluated to predict empty body and hot carcass weight in the Weight dataset [17,21,22,31,32,33,34,35]; Table S2. Equations evaluated to predict empty body chemical composition assuming empty body water is known in the Empty Body dataset [21,22,33]; Table S3. Equations evaluated to predict carcass chemical composition assuming carcass water is known in the Carcass dataset [21,22,33,36]; Table S4. Equations evaluated to predict empty body chemical composition assuming carcass chemical composition is known in the Empty Body-Carcass dataset [21,22]; Literature used to construct the Weight Dataset [64,66,67,68,70,73,85,86,87,99,100,101,102,103,104,105,106,107,108,109,110,111,112,113,114,115,116,117,118,119,120,121,122,123,124,125,126,127,128,129,130,131,132,133,134], Empty Body Dataset [64,66,67,68,70,73,85,86,87,99,102,103,107,109,110,111,112,114,115,116,118,119,120,125,129,130,134,135,136,137], Carcass Dataset [64,67,68,85,86,87,102,111,112,116,125,129,134,136,137], and Empty Body-Carcass Dataset [64,67,68,85,86,87,91,92,102,112,116,129,134,136].

## References

[1] Guiroy P.J., Fox D.G., Tedeschi L.O., Baker M.J., Cravey M.D. (2001). Predicting Individual Feed Requirements of Cattle Fed in Groups. J. Anim. Sci..

[2] Fox D.G., Tedeschi L.O., Tylutki T.P., Russell J.B., Van Amburgh M.E., Chase L.E., Pell A.N., Overton T.R. (2004). The Cornell Net Carbohydrate and Protein System Model for Evaluating Herd Nutrition and Nutrient Excretion. Anim. Feed Sci. Technol..

[3] Tedeschi L.O., Fox D.G., Guiroy P.J. (2004). A Decision Support System to Improve Individual Cattle Management. 1. A Mechanistic, Dynamic Model for Animal Growth. Agric. Syst..

[4] Ferrell C.L., Church D.C. (1988). Energy Metabolism. The Ruminant Animal: Digestive Physiology and Nutrition.

[5] Burrin D.G., Ferrell C.L., Britton R.A., Bauer M.L. (1990). Level of Nutrition and Visceral Organ Size and Metabolic Activity in Sheep. Br. J. Nutr..

[6] Reynolds C.K., Tyrrell H.F., Reynolds P.J. (1991). Effects of Diet Forage-to-Concentrate Ratio and Intake on Energy Metabolism in Growing Beef Heifers: Whole Body Energy and Nitrogen Balance and Visceral Heat Production. J. Nutr..

[7] Koong L.J., Ferrell C.L., Nienaber J.A. (1985). Assessment of Interrelationships among Levels of Intake and Production, Organ Size and Fasting Heat Production in Growing Animals. J. Nutr..

[8] Ferrell C.L., Koong L.J., Nienaber J.A. (1986). Effect of Previous Nutrition on Body Composition and Maintenance Energy Costs of Growing Lambs. Br. J. Nutr..

[9] Ferrell C.L., Crouse J.D., Field R.A., Chant J.L. (1979). Effects of Sex, Diet and Stage of Growth upon Energy Utilization by Lambs. J. Anim. Sci..

[10] Thompson W.R., Meiske J.C., Goodrich R.D., Rust J.R., Byers F.M. (1983). Influence of Body Composition on Energy Requirements of Beef Cows during Winter. J. Anim. Sci..

[11] Tess M.W., Dickerson G.E., Nienaber J.A., Ferrell C.L. (1984). The Effects of Body Composition on Fasting Heat Production in Pigs. J. Anim. Sci..

[12] Solis J.C., Byers F.M., Schelling G.T., Long C.R., Greene L.W. (1988). Maintenance Requirements and Energetic Efficiency of Cows of Different Breed Types. J. Anim. Sci..

[13] DiCostanzo A., Meiske J.C., Plegge S.D., Peters T.M., Goodrich R.D. (1990). Within-Herd Variation in Energy Utilization for Maintenance and Gain in Beef Cows. J. Anim. Sci..

[14] National Research Council (1984). Nutrient Requirements of Beef Cattle.

[15] National Research Council (1976). Nutrient Requirements of Beef Cattle.

[16] National Research Council (2000). Nutrient Requirements of Beef Cattle.

[17] National Academies of Sciences, Engineering, and Medicine (2016). Nutrient Requirements of Beef Cattle.

[18] Blaxter K.L., Rook J.A.F. (1953). The Heat of Combustion of the Tissues of Cattle in Relation to Their Chemical Composition. Br. J. Nutr..

[19] Armsby H.P. (1917). The Nutrition of Farm Animals.

[20] Reid J.T., Bensadoun A., Bull L.S., Burton J.H., Gleeson P.A., Han I.K., Joo Y.D., Johnson D.E., McManus W.R., Paladines O.L. (1968). Some Peculiarities in the Body Composition of Animals. Body Composition in Animals and Man.

[21] Garrett W.N., Hinman N. (1969). Re-Evaluation of the Relationship between Carcass Density and Body Composition of Beef Steers. J. Anim. Sci..

[22] Ferrell C.L., Garrett W.N., Hinman N. (1976). Estimation of Body Composition in Pregnant and Non-Pregnant Heifers. J. Anim. Sci..

[23] National Research Council (1987). Predicting Feed Intake of Food-Producing Animals.

[24] Oltjen J.W., Bywater A.C., Baldwin R.L. (1986). Evaluation of a Model of Beef Cattle Growth and Composition. J. Anim. Sci..

[25] Oltjen J.W., Bywater A.C., Baldwin R.L., Garrett W.N. (1986). Development of a Dynamic Model of Beef Cattle Growth and Composition. J. Anim. Sci..

[26] Perry T.C., Fox D.G. (1997). Predicting Carcass Composition and Individual Feed Requirement in Live Cattle Widely Varying in Body Size. J. Anim. Sci..

[27] McPhee M.J., Oltjen J.W., Fadel J.G., Perry D., Sainz R.D. (2008). Development and Evaluation of Empirical Equations to Interconvert between Twelfth-Rib Fat and Kidney, Pelvic, and Heart Fat Respective Fat Weights and to Predict Initial Conditions of Fat Deposition Models for Beef Cattle. J. Anim. Sci..

[28] Kolath W.H. Feedlot Marker Assisted Management. Proceedings of the Beef Improvement Federation 41st Annual Research Symposium.

[29] Bruns K.W., Pritchard R.H. (2003). Sorting Cattle—A Review.

[30] Cooper R., Klopfenstein T., Milton T., Feuz D. (1999). Feedlot Marketing/Sorting Systems to Reduce Carcass Discounts.

[31] Lofgreen G.P., Hull J.L., Otagaki K.K. (1962). Estimation of Empty Body Weight of Beef Cattle. J. Anim. Sci..

[32] Holzer Z., Levy D. (1969). The Estimation of Empty-Body Weight of Israeli-Friesian and Hereford x Arab Crossbred Bull Calves. Isr. J. Agric. Res..

[33] Gil E.A., Johnson R.R., Cahill V.R., McClure K.E., Klosterman E.W. (1970). An Evaluation of Carcass Specific Volume, Dye Dilution and Empty Body Parameters as Predictors of Beef Carcass Composition over a Wide Range of Fatness. J. Anim. Sci..

[34] Alhassan W.S., Buchanan-Smith J.G., Usborne W.R., Smith G.C., Ashton G.C. (1975). Predicting Empty Body Composition of Cattle from Carcass Weight and Rib Cut Composition. Can. J. Anim. Sci..

[35] Fox D.G., Dockerty T.R., Johnson R.R., Preston R.L. (1976). Relationship of Empty Body Weight to Carcass Weight in Beef Cattle. J. Anim. Sci..

[36] Preston R.L., Vance R.D., Cahill V.R., Kock S.W. (1974). Carcass Specific Gravity and Carcass Composition in Cattle and the Effect of Bone Proportionality on This Relationship. J. Anim. Sci..

[37] Tedeschi L.O. (2006). Assessment of the Adequacy of Mathematical Models. Agric. Syst..

[38] Williams C.B., Keele J.W., Waldo D.R. (1992). A Computer Model to Predict Empty Body Weight in Cattle from Diet and Animal Characteristics. J. Anim. Sci..

[39] Owens F.N., Gill D.R., Secrist D.S., Coleman S.W. (1995). Review of Some Aspects of Growth and Development of Feedlot Cattle. J. Anim. Sci..

[40] Owens F.N., Hicks R.B. (2019). Can Net Energy Values Be Determined from Animal Performance Measurements? A Review of Factors Affecting Application of the California Net Energy System. Transl. Anim. Sci..

[41] Hicks R.B. (1988). Feed Intake of Feedlot Cattle: Prediction Equations and Effect of Restriction. Ph.D. Thesis.

[42] Vasconcelos J.T., Rathmann R.J., Reuter R.R., Leibovich J., McMeniman J.P., Hales K.E., Covey T.L., Miller M.F., Nichols W.T., Galyean M.L. (2008). Effects of Duration of Zilpaterol Hydrochloride Feeding and Days on the Finishing Diet on Feedlot Cattle Performance and Carcass Traits. J. Anim. Sci..

[43] Vasconcelos J.T., Sawyer J.E., Tedeschi L.O., McCollum F.T., Greene L.W. (2009). Effects of Different Growing Diets on Performance, Carcass Characteristics, Insulin Sensitivity, and Accretion of Intramuscular and Subcutaneous Adipose Tissue of Feedlot Cattle. J. Anim. Sci..

[44] MacDonald J., Klopfenstein T., Erickson G.E., Pol K.V. (2007). Changes in Gain through the Feeding Period.

[45] May S.G., Dolezal H.G., Gill D.R., Ray F.K., Buchanan D.S. (1992). Effect of Days Fed, Carcass Grade Traits, and Subcutaneous Fat Removal on Postmortem Muscle Characteristics and Beef Palatability. J. Anim. Sci..

[46] Bruns K.W., Pritchard R.H. (2010). Duration of Feeding: Decision Points. Plains Nutrition Council.

[47] Hedrick H.B., Thompson G.B., Krause G.F. (1969). Comparison of Feedlot Performance and Carcass Characteristics of Half-Sib Bulls, Steers and Heifers. J. Anim. Sci..

[48] Nichols J.R., Ziegler J.H., White J.M., Kesler E.M., Watkins J.L. (1964). Production and Carcass Characteristics of Holstein-Friesian Bulls and Steers Slaughtered at 800 or 1,000 Pounds1. J. Dairy Sci..

[49] Vanderwert W., Berger L.L., McKeith F.K., Baker A.M., Gonyou H.W., Bechtel P.J. (1985). Influence of Zeranol Implants on Growth, Behavior and Carcass Traits in Angus and Limousin Bulls and Steers. J. Anim. Sci..

[50] Coyne J.M., Evans R.D., Berry D.P. (2019). Dressing Percentage and the Differential between Live Weight and Carcass Weight in Cattle Are Influenced by Both Genetic and Non-Genetic Factors. J. Anim. Sci..

[51] Champagne J.R., Carpenter J.W., Hentges J.F., Palmer A.Z., Koger M. (1969). Feedlot Performance and Carcass Characteristics of Young Bulls and Steers Castrated at Four Ages. J. Anim. Sci..

[52] Blanco M., Ripoll G., Delavaud C., Casasús I. (2020). Performance, Carcass and Meat Quality of Young Bulls, Steers and Heifers Slaughtered at a Common Body Weight. Livest. Sci..

[53] Pogorzelska-Przybyłek P., Nogalski Z., Sobczuk-Szul M., Purwin C., Momot M. (2018). Carcass Characteristics of Grass-Fed Crossbred Bulls and Steers Slaughtered at Two Different Ages. Can. J. Anim. Sci..

[54] Price M.A., Jones S.D.M., Mathison G.W., Berg R.T. (1980). The Effects of Increasing Dietary Roughage Level and Slaughter Weight on the Feedlot Performance and Carcass Characteristics of Bulls and Steers. Can. J. Anim. Sci..

[55] Schlickau E.K., Dikeman M.E., Marston T.T., Brethour J., Unruh J.A. (2005). Effects of Early Weaning on Carcass and Ribeye Steak Characteristics of Bulls and Steers. Proceedings of the Cattlemen’s Day.

[56] Chaudhary Z.I., Price M.A., Makarechian M. (1985). Effects of Zeranol on Weight Gain, Bone Growth and Other Carcass Traits in Steers and Bulls. Can. J. Anim. Sci..

[57] Abney C.S. (2004). Feedlot Performance, Carcass and Palatability Traits, as Well as Subsequent Economic Relevance in Calf-Fed and Yearling Holsteins and Angus Steers. Master’s Thesis.

[58] Dean R.A., Holloway J.W., Whiteman J.V., Stephens D.F., Totusek R. (1976). Feedlot Performance of Progeny of Hereford, Hereford × Holstein and Holstein Cows. J. Anim. Sci..

[59] Thonney M.L. (1987). Growth, Feed Efficiency and Variation of Individually Fed Angus, Polled Hereford and Holstein Steers. J. Anim. Sci..

[60] Rust S.R., Abney C.S. Comparison of Dairy versus Beef Steers. https://www.extension.iastate.edu/dairyteam/files/page/files/ComparisonDairyVsBeef_Rust.pdf.

[61] Reid J.T., Wellington G.H., Dunn H.O. (1955). Some Relationships Among the Major Chemical Components of the Bovine Body and Their Application to Nutritional Investigations. J. Dairy Sci..

[62] Murray J.A. (1922). The Chemical Composition of Animal Bodies. J. Agric. Sci..

[63] American Angus Association Genetic Trend EPD/$Value by Birth Year. https://www.angus.org/Nce/GeneticTrends.

[64] Ferrell C.L., Jenkins T.G. (1998). Body Composition and Energy Utilization by Steers of Diverse Genotypes Fed a High-Concentrate Diet during the Finishing Period: I. Angus, Belgian Blue, Hereford, and Piedmontese Sires. J. Anim. Sci..

[65] Kirkpatrick T.J., Wesley K., Pillmore S.L., Cooper K., Tennant T., Lawrence T. (2019). Empty Body Composition and Retained Energy of Jersey Steers Using an Aggressive Implant Strategy. J. Anim. Sci..

[66] Hutcheson J.P., Johnson D.E., Gerken C.L., Morgan J.B., Tatum J.D. (1997). Anabolic Implant Effects on Visceral Organ Mass, Chemical Body Composition, and Estimated Energetic Efficiency in Cloned (Genetically Identical) Beef Steers. J. Anim. Sci..

[67] Rumsey T.S. (1982). Effect of Synovex-S Implants and Kiln Dust on Tissue Gain by Feedlot Beef Steers. J. Anim. Sci..

[68] Rumsey T.S., Elsasser T.H., Kahl S., Moseley W.M., Solomon M.B. (1996). Effects of Synovex-S^®^ and Recombinant Bovine Growth Hormone (Somavubove^®^) on Growth Responses of Steers: I. Performance and Composition of Gain. J. Anim. Sci..

[69] Guiroy P.J., Tedeschi L.O., Fox D.G., Hutcheson J.P. (2002). The Effects of Implant Strategy on Finished Body Weight of Beef Cattle. J. Anim. Sci..

[70] Fortin A., Simpfendorfer S., Reid J.T., Ayala H.J., Anrique R., Kertz A.F. (1980). Effect of Level of Energy Intake and Influence of Breed and Sex on the Chemical Composition of Cattle. J. Anim. Sci..

[71] Steen R.W.J., Kilpatrick D.J. (1995). Effects of Plane of Nutrition and Slaughter Weight on the Carcass Composition of Serially Slaughtered Bulls, Steers and Heifers of Three Breed Crosses. Livest. Prod. Sci..

[72] Conroy S.B., Drennan M.J., McGee M., Keane M.G., Kenny D.A., Berry D.P. (2010). Predicting Beef Carcass Meat, Fat and Bone Proportions from Carcass Conformation and Fat Scores or Hindquarter Dissection. Animal.

[73] Ferrell C.L., Jenkins T.G. (1998). Body Composition and Energy Utilization by Steers of Diverse Genotypes Fed a High-Concentrate Diet during the Finishing Period: II. Angus, Boran, Brahman, Hereford, and Tuli Sires. J. Anim. Sci..

[74] Pringle T.D., Williams S.E., Lamb B.S., Johnson D.D., West R.L. (1997). Carcass Characteristics, the Calpain Proteinase System, and Aged Tenderness of Angus and Brahman Crossbred Steers. J. Anim. Sci..

[75] Crouse J.D., Cundiff L.V., Koch R.M., Koohmaraie M., Seideman S.C. (1989). Comparisons of Bos Indicus and Bos Taurus Inheritance for Carcass Beef Characteristics and Meat Palatability. J. Anim. Sci..

[76] Elzo M.A., Johnson D.D., Wasdin J.G., Driver J.D. (2012). Carcass and Meat Palatability Breed Differences and Heterosis Effects in an Angus–Brahman Multibreed Population. Meat Sci..

[77] Bidner T.D., Wyatt W.E., Humes P.E., Franke D.E., Blouin D.C. (2002). Influence of Brahman-Derivative Breeds and Angus on Carcass Traits, Physical Composition, and Palatability. J. Anim. Sci..

[78] Crockett J.R., Baker F.S., Carpenter J.W., Koger M. (1979). Preweaning, Feedlot and Carcass Characteristics of Calves Sired by Continental, Brahman and Brahman-Derivative Sires in Subtropical Florida. J. Anim. Sci..

[79] Huffman R.D., Williams S.E., Hargrove D.D., Johnson D.D., Marshall T.T. (1990). Effects of Percentage Brahman and Angus Breeding, Age-Season of Feeding and Slaughter End Point on Feedlot Performance and Carcass Characteristics. J. Anim. Sci..

[80] Cole J.W., Ramsey C.B., Hobbs C.S., Temple R.S. (1964). Effects of Type and Breed of British, Zebu, and Dairy Cattle on Production, Palatability, and Composition. III. Percent Wholesale Cuts and Yield of Edible Portion as Determined by Physical and Chemical Analysis. J. Anim. Sci..

[81] Lunt D.K., Smith G.C., Murphey C.E., Savell J.W., Carpenter Z.L., Petersen H.D. (1985). Carcass Characteristics and Composition of Brahman, Angus and Brahman x Angus Steers Fed for Different Times-on-Feed. Meat Sci..

[82] Dikeman M.E., Merkel R.A., Magee W.T. (1977). Effects of Beef-Type on Bone, Fat Trim and Retail Cut Yield and Distribution. J. Anim. Sci..

[83] Judge M.D., Martin T.G., Bramblett V.D., Barton J.A. (1965). Comparison of Dairy and Dual-Purpose Carcasses with Beef-Type Carcasses from Animals of Similar and Younger Ages1. J. Dairy Sci..

[84] Berg R.T., Butterfield R.M. (1968). Growth Patterns of Bovine Muscle, Fat and Bone. J. Anim. Sci..

[85] Coleman S.W., Gallavan R.H., Phillips W.A., Volesky J.D., Rodriguez S. (1995). Silage or Limit-Fed Grain Growing Diets for Steers: II. Empty Body and Carcass Composition. J. Anim. Sci..

[86] Hersom M.J., Horn G.W., Krehbiel C.R., Phillips W.A. (2004). Effect of Live Weight Gain of Steers during Winter Grazing: I. Feedlot Performance, Carcass Characteristics, and Body Composition of Beef Steers. J. Anim. Sci..

[87] McCurdy M.P., Horn G.W., Wagner J.J., Lancaster P.A., Krehbiel C.R. (2010). Effects of Winter Growing Programs on Subsequent Feedlot Performance, Carcass Characteristics, Body Composition, and Energy Requirements of Beef Steers. J. Anim. Sci..

[88] Ferrell C.L. (1988). Contribution of Visceral Organs to Animal Energy Expenditure. J. Anim. Sci..

[89] Drouillard J.S., Klopfenstein T.J., Britton R.A., Bauer M.L., Gramlich S.M., Wester T.J., Ferrell C.L. (1991). Growth, Body Composition, and Visceral Organ Mass and Metabolism in Lambs during and after Metabolizable Protein or Net Energy Restrictions. J. Anim. Sci..

[90] Jenkins T.G., Ferrell C.L. (1997). Changes in Proportions of Empty Body Depots and Constituents for Nine Breeds of Cattle under Various Feed Availabilities. J. Anim. Sci..

[91] Hersom M.J., Krehbiel C.R., Horn G.W. (2004). Effect of Live Weight Gain of Steers during Winter Grazing: II. Visceral Organ Mass, Cellularity, and Oxygen Consumption. J. Anim. Sci..

[92] McCurdy M.P., Krehbiel C.R., Horn G.W., Lancaster P.A., Wagner J.J. (2010). Effects of Winter Growing Program on Visceral Organ Mass, Composition, and Oxygen Consumption of Beef Steers during Growing and Finishing. J. Anim. Sci..

[93] Sainz R.D., Bentley B.E. (1997). Visceral Organ Mass and Cellularity in Growth-Restricted and Refed Beef Steers. J. Anim. Sci..

[94] Jenkins T.G., Ferrell C.L. (1994). Productivity through Weaning of Nine Breeds of Cattle under Varying Feed Availabilities: I. Initial Evaluation. J. Anim. Sci..

[95] Kempster A.J. (1981). Fat Partition and Distribution in the Carcasses of Cattle, Sheep and Pigs: A Review. Meat Sci..

[96] Cianzio D.S., Topel D.G., Whitehurst G.B., Beitz D.C., Self H.L. (1982). Adipose Tissue Growth in Cattle Representing Two Frame Sizes: Distribution among Depots. J. Anim. Sci..

[97] Hankins O.G., Howe P.E. (1946). Estimation of the Composition of Beef Carcasses and Cuts.

[98] Birnie J.W., Agnew R.E., Gordon F.J. (2000). The Influence of Body Condition on the Fasting Energy Metabolism of Nonpregnant, Nonlactating Dairy Cows. J. Dairy Sci..

[99] Basarab J.A., Price M.A., Aalhus J.L., Okine E.K., Snelling W.M., Lyle K.L. (2003). Residual Feed Intake and Body Composition on Young Growing Cattle. Can. J. Anim. Sci..

[100] Gill D.R., Owens F.N., King M.C., Dolezal H.G. (1993). Body Composition of Grazing or Feedlot Steers Differing in Age and Background.

[101] Coleman S.W., Evans B.C., Guenther J.J. (1993). Body and Carcass Composition of Angus and Charolais Steers as Affected by Age and Nutrition. J. Anim. Sci..

[102] Carstens G.E., Johnson D.E., Ellenberger M.A., Tatum J.D. (1991). Physical and Chemical Components of the Empty Body during Compensatory Growth in Beef Steers. J. Anim. Sci..

[103] Baker R.D., Young N.E., Lewis J.A. (1992). The Effect of Diet in Winter on the Body Composition of Young Steers and Subsequent Performance during the Grazing Season. Anim. Prod..

[104] Ponce C.H., Brown M.S., Osterstock J.B., Cole N.A., Lawrence T.E., Soto-Navarro S., MacDonald J., Lambert B.D., Maxwell C. (2014). Effects of Wet Corn Distillers Grains with Solubles on Visceral Organ Mass, Trace Mineral Status, and Polioencephalomalacia Biomarkers of Individually-Fed Cattle. J. Anim. Sci..

[105] Walter L.-A.J., Schmitz A.N., Nichols W.T., Hutcheson J.P., Lawrence T.E. (2018). Live Growth Performance, Carcass Grading Characteristics, and Harvest Yields of Beef Steers Supplemented Zilpaterol Hydrochloride and Offered Ad Libitum or Maintenance Energy Intake. J. Anim. Sci..

[106] Long N.M., Prado-Cooper M.J., Krehbiel C.R., DeSilva U., Wettemann R.P. (2010). Effects of Nutrient Restriction of Bovine Dams during Early Gestation on Postnatal Growth, Carcass and Organ Characteristics, and Gene Expression in Adipose Tissue and Muscle. J. Anim. Sci..

[107] Moallem U., Dahl G.E., Duffey E.K., Capuco A.V., Wood D.L., McLeod K.R., Baldwin R.L., Erdman R.A. (2004). Bovine Somatotropin and Rumen-Undegradable Protein Effects in Prepubertal Dairy Heifers: Effects on Body Composition and Organ and Tissue Weights. J. Dairy Sci..

[108] Krueger W.K., Gutierrez-Bañuelos H., Carstens G.E., Min B.R., Pinchak W.E., Gomez R.R., Anderson R.C., Krueger N.A., Forbes T.D.A. (2010). Effects of Dietary Tannin Source on Performance, Feed Efficiency, Ruminal Fermentation, and Carcass and Non-Carcass Traits in Steers Fed a High-Grain Diet. Anim. Feed Sci. Technol..

[109] Gibb M.J., Baker R.D. (1987). Performance of Young Steers Offered Silage or Thermo-Ammoniated Hay with or without a Fish-Meal Supplement. Anim. Sci..

[110] Gibb M.J., Baker R.D. (1989). Performance and Body Composition of Young Steers given Stack-Ammoniated Hay with or without a Supplement or Untreated Hay with a Supplement. Anim. Sci..

[111] Jesse G.W., Thompson G.B., Clark J.L., Hedrick H.B., Weimer K.G. (1976). Effects of Ration Energy and Slaughter Weight on Composition of Empty Body and Carcass Gain of Beef Cattle. J. Anim. Sci..

[112] Wright I.A., Russel A.J.F. (1991). Changes in the Body Composition of Beef Cattle during Compensatory Growth. Anim. Prod..

[113] Jones S.D.M., Rompala R.E., Jeremiah L.E. (1985). Growth and Composition of the Empty Body in Steers of Different Maturity Types Fed Concentrate or Forage Diets. J. Anim. Sci..

[114] Ferrell C.L., Jenkins T.G. (1985). Energy Utilization by Hereford and Simmental Males and Females. Anim. Sci..

[115] Hammond A.C., Waldo D.R., Rumsey T.S. (1990). Prediction of Body Composition in Holstein Steers Using Urea Space. J. Dairy Sci..

[116] Buckley B.A. (1985). Relationship of Body Composition and Fasting Heat Production in Three Biological Types of Growing Beef Heifers. Ph.D. Dissertation.

[117] Short R.E., Grings E.E., MacNeil M.D., Heitschmidt R.K., Williams C.B., Bennett G.L. (1999). Effects of Sire Growth Potential, Growing-Finishing Strategy, and Time on Feed on Performance, Composition, and Efficiency of Steers. J. Anim. Sci..

[118] Thomas C., Gibbs B.G., Beever D.E., Thurnham B.R. (1988). The Effect of Date of Cut and Barley Substitution on Gain and on the Efficiency of Utilization of Grass Silage by Growing Cattle: 1. Gains in Live Weight and Its Components. Br. J. Nutr..

[119] Waldo D.R., Tyrrell H.F., Capuco A.V., Rexroad C.E. (1997). Components of Growth in Holstein Heifers Fed Either Alfalfa or Corn Silage Diets to Produce Two Daily Gains. J. Dairy Sci..

[120] Baker R.D., Gibb M.J. (1995). The Performance and Changes in Body Composition of Steers Offered Cut Grass or Grazing Following Three Patterns of Nutrition in Winter. Anim. Sci..

[121] Velazco J., Morrill J.L., Kropf D.H., Brandt R.T., Harmon D.L., Preston R.L., Clarenburg R. (1997). The Use of Urea Dilution for Estimation of Carcass Composition of Holstein Steers at 3, 6, 9, and 12 Months of Age. J. Anim. Sci..

[122] Patterson D.C., Steen R.W.J., Kilpatrick D.J. (1995). Growth and Development in Beef Cattle. 1. Direct and Residual Effects of Plane of Nutrition during Early Life on Components of Gain and Food Efficiency. J. Agric. Sci..

[123] Diniz L.L., Filho S.C.V., Campos J.M.S., Valadares R.F.D., Da Silva L.D., Monnerat J.P.I.S., Benedeti P.B., De Oliveira A.S., Pina D.S. (2010). Effects of Castor Meal on the Growth Performance and Carcass Characteristics of Beef Cattle. Asian-Australas. J. Anim. Sci..

[124] Jenkins T.G., Long C.R., Cartwright T.C., Smith G.C. (1981). Characterization of Cattle of a Five—Breed Diallel. IV. Slaughter and Carcass Characters of Serially Slaughtered Bulls. J. Anim. Sci..

[125] Bonilha E.F.M., Branco R.H., Bonilha S.F.M., Araujo F.L., Magnani E., Mercadante M.E.Z. (2013). Body Chemical Composition of Nellore Bulls with Different Residual Feed Intakes. J. Anim. Sci..

[126] Mezzomo R., Paulino P.V.R., Barbosa M.M., da Silva Martins T., Paulino M.F., Alves K.S., Gomes D.I., dos Santos Monnerat J.P.I. (2016). Performance and Carcass Characteristics of Young Cattle Fed with Soybean Meal Treated with Tannins. Anim. Sci. J..

[127] De Almeida V.V.S., Oliveira A.C., Oliveira H.C., Silva R.R., de Lima D.M. (2019). Body Weight Components of Nellore Steers Finished in Tropical Pastures. Acta Sci. Anim. Sci..

[128] Bailey C.B., Lawson J.E. (1989). Carcass and Empty Body Composition of Hereford and Angus Bulls from Lines Selected for Rapid Growth on High-Energy or Low-Energy Diets. Can. J. Anim. Sci..

[129] Kirkpatrick T.J. (2020). The Effect of Growth-Promoting Implants and Feeding Duration on Live Performance and Behavioral Characteristics, Biometric Measurements, Empty Body Composition, and Energy Retention of Serially-Harvested Beef Steers. Master’s Thesis.

[130] Hall J.B., Staigmiller R.B., Bellows R.A., Short R.E., Moseley W.M., Bellows S.E. (1995). Body Composition and Metabolic Profiles Associated with Puberty in Beef Heifers. J. Anim. Sci..

[131] Fidelis H.A., Bonilha S.F.M., Tedeschi L.O., Branco R.H., Cyrillo J.N.S.G., Mercadante M.E.Z. (2017). Residual Feed Intake, Carcass Traits and Meat Quality in Nellore Cattle. Meat Sci..

[132] Fowler M.A., Adeyanju S.A., Burroughs W., Kline E.A. (1970). Net Energy Evaluations of Beef Cattle Rations with and without Stilbestrol. J. Anim. Sci..

[133] Zorzi K., Bonilha S.F.M., Queiroz A.C., Branco R.H., Sobrinho T.L., Duarte M.S. (2013). Meat Quality of Young Nellore Bulls with Low and High Residual Feed Intake. Meat Sci..

[134] Rumsey T.S., Tyrrell H.F., Dinius D.A., Moe P.W., Cross H.R. (1981). Effect of Diethylbestrol on Tissue Gain and Carcass Merit of Feedlot Beef Steers. J. Anim. Sci..

[135] Ryan W.J., Williams I.H., Moir R.J. (1993). Compensatory Growth in Sheep and Cattle. II. Changes in Body Composition and Tissue Weights. Aust. J. Agric. Res..

[136] Patterson D.C., Steen R.W.J. (1995). Growth and Development in Beef Cattle. 2. Direct and Residual Effects of Plane of Nutrition during Early Life on the Chemical Composition of Body Components. J. Agric. Sci..

[137] Bonilha E.F.M., Branco R.H., Bonilha S.F.M., de Araújo F.L., dos S.G. Cyrillo J.N., Magnani E. (2014). Body Chemical Composition, Tissue Deposition Rates and Gain Composition of Young Nellore Cattle Selected for Postweaning Weight. R. Bras. Zootec..

