# Supplementary material for: Assessment of Equations to Predict Body Weight and Chemical Composition in Growing/Finishing Cattle and Effects of Publication Year, Sex, and Breed Type on the Deviation from Observed Values
Source: Animals (Basel). 2022 Dec 15;12(24):3554. doi: 10.3390/ani12243554 (PMC9774178; doi:10.3390/ani12243554)
Supplement: Supplementary file 1 [file animals-12-03554-s001.zip › animals-2065382-supplementary.pdf]

Supplemental Tables

Table S1. Equations evaluated to predict empty body and hot carcass weight in the Weight dataset

Study	Equation[1]
Gil et al., 1970	pEBW, kg = SBW, kg × 0.94 - 7.76
NASEM, 2016	pEBW, kg = SBW, kg × 0.891
Lofgreen et al., 1962	pHCW, kg = (EBW, kg - 31.78) ÷ 1.45
Garrett and Hinman, 1969	pHCW, kg = (EBW, kg - 30.62) ÷ 1.362
Holzer and Levy, 1969 – Eq. 1	pHCW, kg = (EBW, kg - 41.20) ÷ 1.44
Holzer and Levy, 1969 – Eq. 2	pHCW, kg = (EBW, kg - 60.60) ÷ 1.39
Gil et al., 1970	pHCW, kg = EBW, kg × 0.67 – 10.42
Alhassan et al., 1975	pHCW, kg = (EBW, kg - 52.95) ÷ 1.248
Ferrell et al., 1976	pHCW, kg = (EBW, kg - 24.12) ÷ 1.413
Fox et al., 1976	pHCW, kg = (EBW, kg - 40.20) ÷ 1.40

[1]pEBW = predicted empty body weight; SBW = observed shrunk body weight; pHCW = predicted hot carcass weight; EBW = observed empty body weight.

Table S2. Equations evaluated to predict empty body chemical composition assuming empty body water is known in the Empty Body dataset

Study	Equation[1]
Garrett and Hinman, 1969	pEBF, % = 94.32 - 1.266 × EBWA, %
	pEBP, % = (0.1313 × pEBFFDM, % + 0.0331) × 6.25
	pEBE, kcal/g = 1.143 + 0.0847 × EBF
Gil et al., 1970	pEBF, % = 101.3 - 1.34 × EBWA, %
	pEBP, % = 3.37 + 0.20 × EBWA, %
Ferrell et al., 1976	pEBF, % = 96.11 - 1.301 × EBWA, %
	pEBP, % = (0.0362 × EBWA, % + 0.6395) × 6.25
	pEBE, kcal/g = 1.107 + 0.0865 × EBF

[1]pEBF = predicted empty body fat; EBWA = observed empty body water; pEBP = predicted empty body protein; pEBFFDM = predicted empty body fat-free dry matter computed as 100-EBWA-pEBF; pEBE = predicted empty body energy; EBF = observed empty body fat.

Table S3. Equations evaluated to predict carcass chemical composition assuming carcass water is known in the Carcass dataset

Study	Equation[1]
Garrett and Hinman, 1969	pCF, % = 97.76 - 1.337 × CWA, %
	pCP, % = (0.1271 × pCFFDM, % - 0.023) × 6.25
	pCE, kcal/g = 1.170 + 0.0829 × CF
Gil et al., 1970	pCF, % = 99.51 - 1.35 × CWA, %
	pCP, % = 3.98 + 0.22 × CWA, %
Preston et al., 1974	pCF, % = (CWA, % - 72.88) ÷ -0.765
	pCP, % = 3.052 + 0.250 × CWA, %
Ferrell et al., 1976	pCF, % = 97.41 - 1.328× CWA, %
	pCP, % = (0.0396 × CWA, % + 0.3361) × 6.25
	pCE, kcal/g = 1.0577 + 0.0866 × CF

[1]pCF = predicted carcass fat; CWA = observed carcass water; pCP = predicted carcass protein; pCFFDM = predicted carcass fat-free dry matter computed as 100-CWA-pCF; pCE = predicted carcass energy; CF = observed carcass fat.

Table S4. Equations evaluated to predict empty body chemical composition assuming carcass chemical composition is known in the Empty Body-Carcass dataset

Study	Equation[1]
Garrett and Hinman, 1969	pEBWA, % = 3.92 + 0.9702 × CWA, %
	pEBF, % = 0.9246 × CF, % - 0.647
	pEBP, % = (CP ÷ 6.25 × 0.7772 + 0.713) × 6.25
	pEBA, % = 0.844 + 0.6895 × CA, %
	pEBE, kcal/g = 0.94 × CE, kcal/g - 0.003
Ferrell et al., 1976	pEBWA, % = 100 – (0.8488 ×(100 - CWA, %) + 5.087)
	pEBF, % = 0.8431 × CF, % + 1.700
	pEBP, % = (CP ÷ 6.25 × 0.8205 + 0.6304) × 6.25
	pEBA, % = 0.1409 + 0.8630 × CA, %
	pEBE, kcal/g = 0.8438 × CE, kcal/g + 0.3581

[1]pEBWA = predicted empty body water; CWA = observed carcass water; pEBF = predicted empty body fat; CF = observed carcass fat; pEBP = predicted empty body protein; CP = observed carcass protein; pEBA = predicted empty body ash; CA = observed carcass ash; pEBE = predicted empty body energy; CE = observed carcass energy

Literature Cited for Studies Included in Each Dataset

Weight Dataset

1. Basarab, J.A.; Price, M.A.; Aalhus, J.L.; Okine, E.K.; Snelling, W.M.; Lyle, K.L. Residual Feed Intake and Body Composition on Young Growing Cattle. *Can. J. Anim. Sci.* **2003**, *83*, 189–204, doi:10.4141/A02-065.

2. Gill, D.R.; Owens, F.N.; King, M.C.; Dolezal, H.G. *Body Composition of Grazing or Feedlot Steers Differing in Age and Background*; Animal Science Research Report; Oklahoma State University: Stillwater, 1993; pp. 185–190.

3. Ferrell, C.L.; Jenkins, T.G. Body Composition and Energy Utilization by Steers of Diverse Genotypes Fed a High-Concentrate Diet during the Finishing Period: I. Angus, Belgian Blue, Hereford, and Piedmontese Sires. *J. Anim. Sci.* **1998**, *76*, 637–646, doi:10.2527/1998.762637x.

4. Ferrell, C.L.; Jenkins, T.G. Body Composition and Energy Utilization by Steers of Diverse Genotypes Fed a High-Concentrate Diet during the Finishing Period: II. Angus, Boran, Brahman, Hereford, and Tuli Sires. *J. Anim. Sci.* **1998**, *76*, 647–657, doi:10.2527/1998.762647x.

5. Coleman, S.W.; Gallavan, R.H.; Phillips, W.A.; Volesky, J.D.; Rodriguez, S. Silage or Limit-Fed Grain Growing Diets for Steers: II. Empty Body and Carcass Composition. *J. Anim. Sci.* **1995**, *73*, 2621–2630.

6. Coleman, S.W.; Evans, B.C.; Guenther, J.J. Body and Carcass Composition of Angus and Charolais Steers as Affected by Age and Nutrition. *J. Anim. Sci.* **1993**, *71*, 86–95.

7. Carstens, G.E.; Johnson, D.E.; Ellenberger, M.A.; Tatum, J.D. Physical and Chemical Components of the Empty Body during Compensatory Growth in Beef Steers. *J. Anim. Sci.* **1991**, *69*, 3251–3264.

8. Hersom, M.J.; Horn, G.W.; Krehbiel, C.R.; Phillips, W.A. Effect of Live Weight Gain of Steers during Winter Grazing: I. Feedlot Performance, Carcass Characteristics, and Body Composition of Beef Steers. *J. Anim. Sci.* **2004**, *82*, 262–272, doi:/2004.821262x.

9. McCurdy, M.P.; Horn, G.W.; Wagner, J.J.; Lancaster, P.A.; Krehbiel, C.R. Effects of Winter Growing Programs on Subsequent Feedlot Performance, Carcass Characteristics, Body Composition, and Energy Requirements of Beef Steers. *J. Anim. Sci.* **2010**, *88*, 1564–1576, doi:10.2527/jas.2009-2289.

10. Baker, R.D.; Young, N.E.; Lewis, J.A. The Effect of Diet in Winter on the Body Composition of Young Steers and Subsequent Performance during the Grazing Season. *Anim. Prod.* **1992**, *54*, 211–219.

11. Ponce, C.H.; Brown, M.S.; Osterstock, J.B.; Cole, N.A.; Lawrence, T.E.; Soto-Navarro, S.; MacDonald, J.; Lambert, B.D.; Maxwell, C. Effects of Wet Corn Distillers Grains with Solubles on Visceral Organ Mass, Trace Mineral Status, and Polioencephalomalacia Biomarkers of Individually-Fed Cattle. *J Anim Sci* **2014**, *92*, 4034–4046, doi:10.2527/jas.2014-7695.

12. Walter, L.-A.J.; Schmitz, A.N.; Nichols, W.T.; Hutcheson, J.P.; Lawrence, T.E. Live Growth Performance, Carcass Grading Characteristics, and Harvest Yields of Beef Steers Supplemented Zilpaterol Hydrochloride and Offered Ad Libitum or Maintenance Energy Intake. *J Anim Sci* **2018**, *96*, 1688–1703, doi:10.1093/jas/sky105.

13. Long, N.M.; Prado-Cooper, M.J.; Krehbiel, C.R.; DeSilva, U.; Wettemann, R.P. Effects of Nutrient Restriction of Bovine Dams during Early Gestation on Postnatal Growth, Carcass and Organ Characteristics, and Gene Expression in Adipose Tissue and Muscle. *J Anim Sci* **2010**, *88*, 3251–3261, doi:10.2527/jas.2009-2512.

14. Moallem, U.; Dahl, G.E.; Duffey, E.K.; Capuco, A.V.; Wood, D.L.; McLeod, K.R.; Baldwin, R.L.; Erdman, R.A. Bovine Somatotropin and Rumen-Undegradable Protein Effects in Prepubertal Dairy Heifers: Effects on Body Composition and Organ and Tissue Weights*. *Journal of Dairy Science* **2004**, *87*, 3869–3880, doi:10.3168/jds.S0022-0302(04)73526-2.

15. Krueger, W.K.; Gutierrez-Bañuelos, H.; Carstens, G.E.; Min, B.R.; Pinchak, W.E.; Gomez, R.R.; Anderson, R.C.; Krueger, N.A.; Forbes, T.D.A. Effects of Dietary Tannin Source on Performance, Feed Efficiency, Ruminal Fermentation, and Carcass and Non-Carcass Traits in Steers Fed a High-Grain Diet. *Animal Feed Science and Technology* **2010**, *159*, 1–9, doi:10.1016/j.anifeedsci.2010.05.003.

16. Gibb, M.J.; Baker, R.D. Performance of Young Steers Offered Silage or Thermo-Ammoniated Hay with or without a Fish-Meal Supplement. *Animal Science* **1987**, *45*, 371–381, doi:10.1017/S0003356100002865.

17. Gibb, M.J.; Baker, R.D. Performance and Body Composition of Young Steers given Stack-Ammoniated Hay with or without a Supplement or Untreated Hay with a Supplement. *Animal Science* **1989**, *48*, 341–351, doi:10.1017/S0003356100040332.

18. Jesse, G.W.; Thompson, G.B.; Clark, J.L.; Hedrick, H.B.; Weimer, K.G. Effects of Ration Energy and Slaughter Weight on Composition of Empty Body and Carcass Gain of Beef Cattle. *J. Anim. Sci.* **1976**, *43*, 418–425.

19. Wright, I.A.; Russel, A.J.F. Changes in the Body Composition of Beef Cattle during Compensatory Growth. *Anim. Prod.* **1991**, *52*, 105–113.

20. Jones, S.D.M.; Rompala, R.E.; Jeremiah, L.E. Growth and Composition of the Empty Body in Steers of Different Maturity Types Fed Concentrate or Forage Diets. *J. Anim. Sci.* **1985**, *60*, 427–433.

21. Ferrell, C.L.; Jenkins, T.G. Energy Utilization by Hereford and Simmental Males and Females. *Animal Science* **1985**, *41*, 53–61, doi:10.1017/S0003356100017542.

22. A. C. Hammond; D. R. Waldo; T. S. Rumsey Prediction of Body Composition in Holstein Steers Using Urea Space. *J. Dairy Sci.* **1990**, *73*, 3141–3145.

23. Buckley, B.A. Relationship of Body Composition and Fasting Heat Production in Three Biological Types of Growing Beef Heifers. Ph.D. Dissertation, University of Nebraska, Lincoln: Lincoln, NE, 1985.

24. Hutcheson, J.P.; Johnson, D.E.; Gerken, C.L.; Morgan, J.B.; Tatum, J.D. Anabolic Implant Effects on Visceral Organ Mass, Chemical Body Composition, and Estimated Energetic Efficiency in Cloned (Genetically Identical) Beef Steers. *Journal of Animal Science* **1997**, *75*, 2620, doi:10.2527/1997.75102620x.

25. Rumsey, T.S. Effect of Synovex-S Implants and Kiln Dust on Tissue Gain by Feedlot Beef Steers. *J. Anim. Sci.* **1982**, *54*, 1030–1039.

26. Rumsey, T.S.; Elsasser, T.H.; Kahl, S.; Moseley, W.M.; Solomon, M.B. Effects of Synovex-S® and Recombinant Bovine Growth Hormone (Somavubove®) on Growth Responses of Steers: I. Performance and Composition of Gain. *J Anim Sci* **1996**, *74*, 2917–2928, doi:10.2527/1996.74122917x.

27. Short, R.E.; Grings, E.E.; MacNeil, M.D.; Heitschmidt, R.K.; Williams, C.B.; Bennett, G.L. Effects of Sire Growth Potential, Growing-Finishing Strategy, and Time on Feed on Performance, Composition, and Efficiency of Steers. *J. Anim. Sci.* **1999**, *77*, 2406–2417.

28. Thomas, C.; Gibbs, B.G.; Beever, D.E.; Thurnham, B.R. The Effect of Date of Cut and Barley Substitution on Gain and on the Efficiency of Utilization of Grass Silage by Growing Cattle: 1. Gains in Live Weight and Its Components. *British Journal of Nutrition* **1988**, *60*, 297–306, doi:10.1079/BJN19880101.

29. Waldo, D.R.; Tyrrell, H.F.; Capuco, A.V.; Rexroad, C.E. Components of Growth in Holstein Heifers Fed Either Alfalfa or Corn Silage Diets to Produce Two Daily Gains. *Journal of Dairy Science* **1997**, *80*, 1674–1684, doi:10.3168/jds.S0022-0302(97)76099-5.

30. Baker, R.D.; Gibb, M.J. The Performance and Changes in Body Composition of Steers Offered Cut Grass or Grazing Following Three Patterns of Nutrition in Winter. *Animal Science* **1995**, *60*, 419–427, doi:10.1017/S1357729800013291.

31. Velazco, J.; Morrill, J.L.; Kropf, D.H.; Brandt, R.T.; Harmon, D.L.; Preston, R.L.; Clarenburg, R. The Use of Urea Dilution for Estimation of Carcass Composition of Holstein Steers at 3, 6, 9, and 12 Months of Age. *Journal of Animal Science* **1997**, *75*, 139, doi:10.2527/1997.751139x.

32. Patterson, D.C.; Steen, R.W.J.; Kilpatrick, D.J. Growth and Development in Beef Cattle. 1. Direct and Residual Effects of Plane of Nutrition during Early Life on Components of

Gain and Food Efficiency. *J. Agric. Sci.* **1995**, *124*, 91–100, doi:10.1017/S002185960007129X.

33. Diniz, L.L.; Filho, S.C.V.; Campos, J.M.S.; Valadares, R.F.D.; Da Silva, L.D.; Monnerat, J.P.I.S.; Benedeti, P.B.; De Oliveira, A.S.; Pina, D.S. Effects of Castor Meal on the Growth Performance and Carcass Characteristics of Beef Cattle. *Asian-Australasian Journal of Animal Sciences* **2010**, *23*, 1308–1318, doi:10.5713/ajas.2010.10041.

34. Jenkins, T.G.; Long, C.R.; Cartwright, T.C.; Smith, G.C. Characterization of Cattle of a Five - Breed Diallel. IV. Slaughter and Carcass Characters of Serially Slaughtered Bulls. *Journal of Animal Science* **1981**, *53*, 62–79, doi:10.2527/jas1981.53162x.

35. Fortin, A.; Simpfendorfer, S.; Reid, J.T.; Ayala, H.J.; Anrique, R.; Kertz, A.F. Effect of Level of Energy Intake and Influence of Breed and Sex on the Chemical Composition of Cattle. *J. Anim. Sci.* **1980**, *51*, 604–614.

36. Bonilha, E.F.M.; Branco, R.H.; Bonilha, S.F.M.; Araujo, F.L.; Magnani, E.; Mercadante, M.E.Z. Body Chemical Composition of Nellore Bulls with Different Residual Feed Intakes. *J Anim Sci* **2013**, *91*, 3457–3464, doi:10.2527/jas.2012-5437.

37. Mezzomo, R.; Paulino, P.V.R.; Barbosa, M.M.; da Silva Martins, T.; Paulino, M.F.; Alves, K.S.; Gomes, D.I.; dos Santos Monnerat, J.P.I. Performance and Carcass Characteristics of Young Cattle Fed with Soybean Meal Treated with Tannins. *Animal Science Journal* **2016**, *87*, 775–782, doi:10.1111/asj.12486.

38. Rumsey, T.S.; Tyrrell, H.F.; Dinius, D.A.; Moe, P.W.; Cross, H.R. Effect of Diethylbestrol on Tissue Gain and Carcass Merit of Feedlot Beef Steers. *J. Anim. Sci.* **1981**, *53*, 589–600.

39. Almeida, V.V.S. de; Oliveira, A.C.; Oliveira, H.C.; Silva, R.R.; Lima, D.M. de Body Weight Components of Nellore Steers Finished in Tropical Pastures. *Acta Sci., Anim. Sci.* **2019**, *41*, doi:10.4025/actascianimsci.v41i1.39005.

40. Bailey, C.B.; Lawson, J.E. Carcass and Empty Body Composition of Hereford and Angus Bulls from Lines Selected for Rapid Growth on High-Energy or Low-Energy Diets. *Can. J. Anim. Sci.* **1989**, *69*, 583–594, doi:10.4141/cjas89-070.

41. Kirkpatrick, T.J. The Effect of Growth-Promoting Implants and Feeding Duration on Live Performance and Behavioral Characteristics, Biometric Measurements, Empty Body Composition, and Energy Retention of Serially-Harvested Beef Steers. Thesis, 2020.

42. Hall, J.B.; Staigmiller, R.B.; Bellows, R.A.; Short, R.E.; Moseley, W.M.; Bellows, S.E. Body Composition and Metabolic Profiles Associated with Puberty in Beef Heifers. *J. Anim. Sci.* **1995**, *73*, 3409–3420.

43. Fidelis, H.A.; Bonilha, S.F.M.; Tedeschi, L.O.; Branco, R.H.; Cyrillo, J.N.S.G.; Mercadante, M.E.Z. Residual Feed Intake, Carcass Traits and Meat Quality in Nellore Cattle. *Meat Science* **2017**, *128*, 34–39, doi:10.1016/j.meatsci.2017.02.004.

44. Fowler, M.A.; Adeyanju, S.A.; Burroughs, W.; Kline, E.A. Net Energy Evaluations of Beef Cattle Rations with and without Stilbestrol. *Journal of Animal Science* **1970**, *30*, 291–296, doi:10.2527/jas1970.302291x.

45. Zorzi, K.; Bonilha, S.F.M.; Queiroz, A.C.; Branco, R.H.; Sobrinho, T.L.; Duarte, M.S. Meat Quality of Young Nellore Bulls with Low and High Residual Feed Intake. *Meat Science* **2013**, *93*, 593–599, doi:10.1016/j.meatsci.2012.11.030.

Empty Body Dataset

1. Basarab, J.A.; Price, M.A.; Aalhus, J.L.; Okine, E.K.; Snelling, W.M.; Lyle, K.L. Residual Feed Intake and Body Composition on Young Growing Cattle. *Can. J. Anim. Sci.* **2003**, *83*, 189–204, doi:10.4141/A02-065.

2. Ferrell, C.L.; Jenkins, T.G. Body Composition and Energy Utilization by Steers of Diverse Genotypes Fed a High-Concentrate Diet during the Finishing Period: I. Angus, Belgian Blue, Hereford, and Piedmontese Sires. *J. Anim. Sci.* **1998**, *76*, 637–646, doi:10.2527/1998.762637x.

3. Ferrell, C.L.; Jenkins, T.G. Body Composition and Energy Utilization by Steers of Diverse Genotypes Fed a High-Concentrate Diet during the Finishing Period: II. Angus, Boran, Brahman, Hereford, and Tuli Sires. *J. Anim. Sci.* **1998**, *76*, 647–657, doi:10.2527/1998.762647x.

4. Coleman, S.W.; Gallavan, R.H.; Phillips, W.A.; Volesky, J.D.; Rodriguez, S. Silage or Limit-Fed Grain Growing Diets for Steers: II. Empty Body and Carcass Composition. *J. Anim. Sci.* **1995**, *73*, 2621–2630.

5. Carstens, G.E.; Johnson, D.E.; Ellenberger, M.A.; Tatum, J.D. Physical and Chemical Components of the Empty Body during Compensatory Growth in Beef Steers. *J. Anim. Sci.* **1991**, *69*, 3251–3264.

6. Hersom, M.J.; Horn, G.W.; Krehbiel, C.R.; Phillips, W.A. Effect of Live Weight Gain of Steers during Winter Grazing: I. Feedlot Performance, Carcass Characteristics, and Body Composition of Beef Steers. *J. Anim. Sci.* **2004**, *82*, 262–272, doi:/2004.821262x.

7. McCurdy, M.P.; Horn, G.W.; Wagner, J.J.; Lancaster, P.A.; Krehbiel, C.R. Effects of Winter Growing Programs on Subsequent Feedlot Performance, Carcass Characteristics, Body Composition, and Energy Requirements of Beef Steers. *J. Anim. Sci.* **2010**, *88*, 1564–1576, doi:10.2527/jas.2009-2289.

8. Baker, R.D.; Young, N.E.; Lewis, J.A. The Effect of Diet in Winter on the Body Composition of Young Steers and Subsequent Performance during the Grazing Season. *Anim. Prod.* **1992**, *54*, 211–219.

9. Moallem, U.; Dahl, G.E.; Duffey, E.K.; Capuco, A.V.; Wood, D.L.; McLeod, K.R.; Baldwin, R.L.; Erdman, R.A. Bovine Somatotropin and Rumen-Undegradable Protein Effects in Prepubertal Dairy Heifers: Effects on Body Composition and Organ and Tissue Weights*. *Journal of Dairy Science* **2004**, *87*, 3869–3880, doi:10.3168/jds.S0022-0302(04)73526-2.

10. Gibb, M.J.; Baker, R.D. Performance of Young Steers Offered Silage or Thermo-Ammoniated Hay with or without a Fish-Meal Supplement. *Animal Science* **1987**, *45*, 371–381, doi:10.1017/S0003356100002865.

11. Gibb, M.J.; Baker, R.D. Performance and Body Composition of Young Steers given Stack-Ammoniated Hay with or without a Supplement or Untreated Hay with a Supplement. *Animal Science* **1989**, *48*, 341–351, doi:10.1017/S0003356100040332.

12. Jesse, G.W.; Thompson, G.B.; Clark, J.L.; Hedrick, H.B.; Weimer, K.G. Effects of Ration Energy and Slaughter Weight on Composition of Empty Body and Carcass Gain of Beef Cattle. *J. Anim. Sci.* **1976**, *43*, 418–425.

13. Wright, I.A.; Russel, A.J.F. Changes in the Body Composition of Beef Cattle during Compensatory Growth. *Anim. Prod.* **1991**, *52*, 105–113.

14. Ferrell, C.L.; Jenkins, T.G. Energy Utilization by Hereford and Simmental Males and Females. *Animal Science* **1985**, *41*, 53–61, doi:10.1017/S0003356100017542.

15. A. C. Hammond; D. R. Waldo; T. S. Rumsey Prediction of Body Composition in Holstein Steers Using Urea Space. *J. Dairy Sci.* **1990**, *73*, 3141–3145.

16. Buckley, B.A. Relationship of Body Composition and Fasting Heat Production in Three Biological Types of Growing Beef Heifers. Ph.D. Dissertation, University of Nebraska, Lincoln: Lincoln, NE, 1985.

17. Hutcheson, J.P.; Johnson, D.E.; Gerken, C.L.; Morgan, J.B.; Tatum, J.D. Anabolic Implant Effects on Visceral Organ Mass, Chemical Body Composition, and Estimated Energetic Efficiency in Cloned (Genetically Identical) Beef Steers. *Journal of Animal Science* **1997**, *75*, 2620, doi:10.2527/1997.75102620x.

18. Rumsey, T.S. Effect of Synovex-S Implants and Kiln Dust on Tissue Gain by Feedlot Beef Steers. *J. Anim. Sci.* **1982**, *54*, 1030–1039.

19. Rumsey, T.S.; Elsasser, T.H.; Kahl, S.; Moseley, W.M.; Solomon, M.B. Effects of Synovex-S® and Recombinant Bovine Growth Hormone (Somavubove®) on Growth Responses of Steers: I. Performance and Composition of Gain. *J Anim Sci* **1996**, *74*, 2917–2928, doi:10.2527/1996.74122917x.

20. Thomas, C.; Gibbs, B.G.; Beever, D.E.; Thurnham, B.R. The Effect of Date of Cut and Barley Substitution on Gain and on the Efficiency of Utilization of Grass Silage by Growing Cattle: 1. Gains in Live Weight and Its Components. *British Journal of Nutrition* **1988**, *60*, 297–306, doi:10.1079/BJN19880101.

21. Waldo, D.R.; Tyrrell, H.F.; Capuco, A.V.; Rexroad, C.E. Components of Growth in Holstein Heifers Fed Either Alfalfa or Corn Silage Diets to Produce Two Daily Gains. *Journal of Dairy Science* **1997**, *80*, 1674–1684, doi:10.3168/jds.S0022-0302(97)76099-5.

22. Baker, R.D.; Gibb, M.J. The Performance and Changes in Body Composition of Steers Offered Cut Grass or Grazing Following Three Patterns of Nutrition in Winter. *Animal Science* **1995**, *60*, 419–427, doi:10.1017/S1357729800013291.

23. Fortin, A.; Simpfendorfer, S.; Reid, J.T.; Ayala, H.J.; Anrique, R.; Kertz, A.F. Effect of Level of Energy Intake and Influence of Breed and Sex on the Chemical Composition of Cattle. *J. Anim. Sci.* **1980**, *51*, 604–614.

24. Bonilha, E.F.M.; Branco, R.H.; Bonilha, S.F.M.; Araujo, F.L.; Magnani, E.; Mercadante, M.E.Z. Body Chemical Composition of Nellore Bulls with Different Residual Feed Intakes. *J Anim Sci* **2013**, *91*, 3457–3464, doi:10.2527/jas.2012-5437.

25. Rumsey, T.S.; Tyrrell, H.F.; Dinius, D.A.; Moe, P.W.; Cross, H.R. Effect of Diethylbestrol on Tissue Gain and Carcass Merit of Feedlot Beef Steers. *J. Anim. Sci.* **1981**, *53*, 589–600.

26. Kirkpatrick, T.J. The Effect of Growth-Promoting Implants and Feeding Duration on Live Performance and Behavioral Characteristics, Biometric Measurements, Empty Body Composition, and Energy Retention of Serially-Harvested Beef Steers. Thesis, 2020.

27. Hall, J.B.; Staigmiller, R.B.; Bellows, R.A.; Short, R.E.; Moseley, W.M.; Bellows, S.E. Body Composition and Metabolic Profiles Associated with Puberty in Beef Heifers. *J. Anim. Sci.* **1995**, *73*, 3409–3420.

28. Ryan, W.J.; Williams, I.H.; Moir, R.J. Compensatory Growth in Sheep and Cattle. II. Changes in Body Composition and Tissue Weights. *Aust. J. Agric. Res.* **1993**, *44*, 1623–1633, doi:10.1071/ar9931623.

29. Patterson, D.C.; Steen, R.W.J. Growth and Development in Beef Cattle. 2. Direct and Residual Effects of Plane of Nutrition during Early Life on the Chemical Composition of Body Components. *J. Agric. Sci.* **1995**, *124*, 101–111, doi:10.1017/S0021859600071306.

30. Bonilha, E.F.M.; Branco, R.H.; Bonilha, S.F.M.; Araújo, F.L. de; Cyrillo, J.N. dos S.G.; Magnani, E. Body Chemical Composition, Tissue Deposition Rates and Gain Composition of Young Nellore Cattle Selected for Postweaning Weight. *R. Bras. Zootec.* **2014**, *43*, 175–182, doi:10.1590/S1516-35982014000400003.

Carcass Dataset

1. Ferrell, C.L.; Jenkins, T.G. Body Composition and Energy Utilization by Steers of Diverse Genotypes Fed a High-Concentrate Diet during the Finishing Period: I. Angus, Belgian Blue, Hereford, and Piedmontese Sires. *J. Anim. Sci.* **1998**, *76*, 637–646, doi:10.2527/1998.762637x.

2. Coleman, S.W.; Gallavan, R.H.; Phillips, W.A.; Volesky, J.D.; Rodriguez, S. Silage or Limit-Fed Grain Growing Diets for Steers: II. Empty Body and Carcass Composition. *J. Anim. Sci.* **1995**, *73*, 2621–2630.

3. Carstens, G.E.; Johnson, D.E.; Ellenberger, M.A.; Tatum, J.D. Physical and Chemical Components of the Empty Body during Compensatory Growth in Beef Steers. *J. Anim. Sci.* **1991**, *69*, 3251–3264.

4. Hersom, M.J.; Horn, G.W.; Krehbiel, C.R.; Phillips, W.A. Effect of Live Weight Gain of Steers during Winter Grazing: I. Feedlot Performance, Carcass Characteristics, and Body Composition of Beef Steers. *J. Anim. Sci.* **2004**, *82*, 262–272, doi:/2004.821262x.

5. McCurdy, M.P.; Horn, G.W.; Wagner, J.J.; Lancaster, P.A.; Krehbiel, C.R. Effects of Winter Growing Programs on Subsequent Feedlot Performance, Carcass Characteristics, Body Composition, and Energy Requirements of Beef Steers. *J. Anim. Sci.* **2010**, *88*, 1564–1576, doi:10.2527/jas.2009-2289.

6. Jesse, G.W.; Thompson, G.B.; Clark, J.L.; Hedrick, H.B.; Weimer, K.G. Effects of Ration Energy and Slaughter Weight on Composition of Empty Body and Carcass Gain of Beef Cattle. *J. Anim. Sci.* **1976**, *43*, 418–425.

7. Wright, I.A.; Russel, A.J.F. Changes in the Body Composition of Beef Cattle during Compensatory Growth. *Anim. Prod.* **1991**, *52*, 105–113.

8. Buckley, B.A. Relationship of Body Composition and Fasting Heat Production in Three Biological Types of Growing Beef Heifers. Ph.D. Dissertation, University of Nebraska, Lincoln: Lincoln, NE, 1985.

9. Rumsey, T.S. Effect of Synovex-S Implants and Kiln Dust on Tissue Gain by Feedlot Beef Steers. *J. Anim. Sci.* **1982**, *54*, 1030–1039.

10. Rumsey, T.S.; Elsasser, T.H.; Kahl, S.; Moseley, W.M.; Solomon, M.B. Effects of Synovex-S® and Recombinant Bovine Growth Hormone (Somavubove®) on Growth Responses of Steers: I. Performance and Composition of Gain. *J Anim Sci* **1996**, *74*, 2917–2928, doi:10.2527/1996.74122917x.

11. Patterson, D.C.; Steen, R.W.J. Growth and Development in Beef Cattle. 2. Direct and Residual Effects of Plane of Nutrition during Early Life on the Chemical Composition of Body Components. *J. Agric. Sci.* **1995**, *124*, 101–111, doi:10.1017/S0021859600071306.

12. Bonilha, E.F.M.; Branco, R.H.; Bonilha, S.F.M.; Araujo, F.L.; Magnani, E.; Mercadante, M.E.Z. Body Chemical Composition of Nellore Bulls with Different Residual Feed Intakes. *J Anim Sci* **2013**, *91*, 3457–3464, doi:10.2527/jas.2012-5437.

13. Bonilha, E.F.M.; Branco, R.H.; Bonilha, S.F.M.; Araújo, F.L. de; Cyrillo, J.N. dos S.G.; Magnani, E. Body Chemical Composition, Tissue Deposition Rates and Gain Composition of Young Nellore Cattle Selected for Postweaning Weight. *R. Bras. Zootec.* **2014**, *43*, 175–182, doi:10.1590/S1516-35982014000400003.

14. Rumsey, T.S.; Tyrrell, H.F.; Dinius, D.A.; Moe, P.W.; Cross, H.R. Effect of Diethylbestrol on Tissue Gain and Carcass Merit of Feedlot Beef Steers. *J. Anim. Sci.* **1981**, *53*, 589–600.

15. Kirkpatrick, T.J. The Effect of Growth-Promoting Implants and Feeding Duration on Live Performance and Behavioral Characteristics, Biometric Measurements, Empty Body Composition, and Energy Retention of Serially-Harvested Beef Steers. Thesis, 2020.

Empty Body-Carcass Dataset

1. Ferrell, C.L.; Jenkins, T.G. Body Composition and Energy Utilization by Steers of Diverse Genotypes Fed a High-Concentrate Diet during the Finishing Period: I. Angus, Belgian Blue, Hereford, and Piedmontese Sires. *J. Anim. Sci.* **1998**, *76*, 637–646, doi:10.2527/1998.762637x.

2. Coleman, S.W.; Gallavan, R.H.; Phillips, W.A.; Volesky, J.D.; Rodriguez, S. Silage or Limit-Fed Grain Growing Diets for Steers: II. Empty Body and Carcass Composition. *J. Anim. Sci.* **1995**, *73*, 2621–2630.

3. Carstens, G.E.; Johnson, D.E.; Ellenberger, M.A.; Tatum, J.D. Physical and Chemical Components of the Empty Body during Compensatory Growth in Beef Steers. *J. Anim. Sci.* **1991**, *69*, 3251–3264.

4. Hersom, M.J.; Horn, G.W.; Krehbiel, C.R.; Phillips, W.A. Effect of Live Weight Gain of Steers during Winter Grazing: I. Feedlot Performance, Carcass Characteristics, and Body Composition of Beef Steers. *J. Anim. Sci.* **2004**, *82*, 262–272, doi:/2004.821262x.

5. Hersom, M.J.; Krehbiel, C.R.; Horn, G.W. Effect of Live Weight Gain of Steers during Winter Grazing: II. Visceral Organ Mass, Cellularity, and Oxygen Consumption. *J. Anim. Sci.* **2004**, *82*, 184–197.

6. McCurdy, M.P.; Horn, G.W.; Wagner, J.J.; Lancaster, P.A.; Krehbiel, C.R. Effects of Winter Growing Programs on Subsequent Feedlot Performance, Carcass Characteristics, Body Composition, and Energy Requirements of Beef Steers. *J. Anim. Sci.* **2010**, *88*, 1564–1576, doi:10.2527/jas.2009-2289.

7. McCurdy, M.P.; Krehbiel, C.R.; Horn, G.W.; Lancaster, P.A.; Wagner, J.J. Effects of Winter Growing Program on Visceral Organ Mass, Composition, and Oxygen Consumption of Beef Steers during Growing and Finishing. *J. Anim. Sci.* **2010**, *88*, 1554–1563, doi:10.2527/jas.2009-2415.

8. Wright, I.A.; Russel, A.J.F. Changes in the Body Composition of Beef Cattle during Compensatory Growth. *Anim. Prod.* **1991**, *52,* 105–113.

9. Buckley, B.A. Relationship of Body Composition and Fasting Heat Production in Three Biological Types of Growing Beef Heifers. Ph.D. Dissertation, University of Nebraska, Lincoln: Lincoln, NE, 1985.

10. Rumsey, T.S. Effect of Synovex-S Implants and Kiln Dust on Tissue Gain by Feedlot Beef Steers. *J. Anim. Sci.* **1982**, *54*, 1030–1039.

11. Rumsey, T.S.; Elsasser, T.H.; Kahl, S.; Moseley, W.M.; Solomon, M.B. Effects of Synovex-S® and Recombinant Bovine Growth Hormone (Somavubove®) on Growth Responses of Steers: I. Performance and Composition of Gain. *J Anim Sci* **1996**, *74,* 2917–2928, doi:10.2527/1996.74122917x.

12. Patterson, D.C.; Steen, R.W.J. Growth and Development in Beef Cattle. 2. Direct and Residual Effects of Plane of Nutrition during Early Life on the Chemical Composition of Body Components. *J. Agric. Sci.* **1995**, *124*, 101–111, doi:10.1017/S0021859600071306.

13. Rumsey, T.S.; Tyrrell, H.F.; Dinius, D.A.; Moe, P.W.; Cross, H.R. Effect of Diethylbestrol on Tissue Gain and Carcass Merit of Feedlot Beef Steers. *J. Anim. Sci.* **1981**, *53*, 589–600.

14. Kirkpatrick, T.J. The Effect of Growth-Promoting Implants and Feeding Duration on Live Performance and Behavioral Characteristics, Biometric Measurements, Empty Body Composition, and Energy Retention of Serially-Harvested Beef Steers. Thesis, 2020.
